# Cu-HKUST-1 and Hydroxyapatite–The Interface of Two Worlds toward the Design of Functional Materials Dedicated to Bone Tissue Regeneration

**DOI:** 10.1021/acsbiomaterials.3c00594

**Published:** 2023-08-01

**Authors:** Marzena Fandzloch, Weronika Bodylska, Joanna Trzcińska-Wencel, Patrycja Golińska, Katarzyna Roszek, Joanna Wiśniewska, Michał Bartmański, Agnieszka Lewińska, Anna Jaromin

**Affiliations:** † Institute of Low Temperature and Structure Research, Polish Academy of Sciences, Okólna 2, 50-422 Wrocław, Poland; ‡ Faculty of Biological and Veterinary Sciences, Nicolaus Copernicus University in Toruń, Lwowska 1, 87-100 Toruń, Poland; § Faculty of Chemistry, Nicolaus Copernicus University in Toruń, Gagarina 7, 87-100 Toruń, Poland; ∥ Faculty of Mechanical Engineering and Ship Technology, Gdańsk University of Technology, Gabriela Narutowicza 11/12, 80-233 Gdańsk, Poland; ⊥ Faculty of Chemistry, University of Wrocław, F. Joliot-Curie 14, 50-383 Wrocław, Poland; # Department of Lipids and Liposomes, Faculty of Biotechnology, University of Wrocław, F. Joliot-Curie 14a, 50-383 Wrocław, Poland

**Keywords:** Cu-HKUST-1, hydroxyapatite, bone tissue regeneration, human dermal fibroblasts, nanomechanical properties, antibacterial activity, biomimetic bone-substitution materials

## Abstract

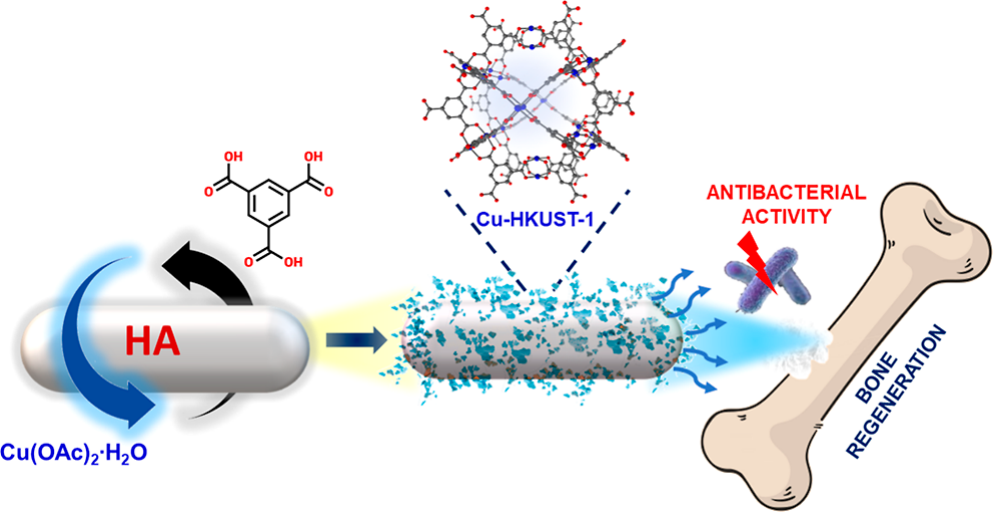

A novel composite based on biocompatible hydroxyapatite (HA) nanoparticles and Cu-HKUST-1 (Cu-HKUST-1@HA) has been prepared following a layer-by-layer strategy. Cu-HKUST-1 was carefully selected from a group of four Cu-based metal–organic frameworks as the material with the most promising antimicrobial activity. The formation of a colloidal Cu-HKUST-1 layer on HA nanoparticles was confirmed by various techniques, *e.g.*, infrared spectroscopy, powder X-ray diffraction, N_2_ sorption, transmission electron microscopy imaging, electron paramagnetic resonance, and X-ray absorption spectroscopy. Importantly, such a Cu-HKUST-1 layer significantly improved the nanomechanical properties of the composite, with Young’s modulus equal to that of human cortical bone (13.76 GPa). At the same time, Cu-HKUST-1@HA has maintained the negative zeta potential (−16.3 mV in pH 7.4) and revealed biocompatibility toward human dermal fibroblasts up to a concentration of 1000 μg/mL, without inducing *ex vivo* hemolysis. Chemical stability studies of the composite over 21 days in a buffer-simulated physiological fluid allowed a detailed understanding of the transformations that the Cu-HKUST-1@HA undergoes over time. Finally, it has been confirmed that the Cu-HKUST-1 layer provides antibacterial properties to HA, and the synergism reached in this way makes it promising for bone tissue regeneration.

One of the biomaterials widely used for bio-related applications is hydroxyapatite (HA). Naturally, HA is produced by the biomineralization process in living cells; however, it can be easily obtained chemically with a unique Ca/P ratio of 1.67, similar to apatite in human bones.^1^ On the other hand, also HA with various compositional and structural defects, including calcium and hydroxyl deficiencies and several ionic substitutions, *e.g.*, CO_3_^2–^ replacing PO_4_^3–^ groups (B-type) or OH^–^ ions (A-type), offer an interesting approach to the preparation of biomimetic bone-substitution materials.^2,3^ Thus, chemically synthesized HA is nowadays extensively used in bone treatments, controlled drug release, and as coatings on orthopedic or dental implants due to its high biocompatibility and good mechanical properties. High bond strength and an elastic modulus value close to that of the bone represent just a few of them.^4,5^ Nevertheless, bacterial infections of medical implants are one of the most common causes of failure of implant therapy.^6^ Implanted biomaterials can act as an avenue for both bacterial contamination and colonization toward the development of osteomyelitis. If the conditions are favorable, bacteria create an initial attachment to the surface. Because biomaterials do not elicit an antiphagocytic reaction toward bacteria after adhesion, they are able to multiply and colonize freely on implant surfaces.^7^

Consequently, the search for multifunctional HA-based composite materials with antibacterial properties is a required approach. Various strategies are used to provide HA with antibacterial properties. These include, for example, doping with silver nanoparticles (Ag/HA)^8^ or mixing it with copper (Ag–Cu/HA).^9^ HA doped with silver may also be used as a component for more complex materials containing polymers, such as chitosan.^10−13^ For these composites, antimicrobial activity against *Escherichia coli* and *Staphylococcus aureus* has been reported. It is worth noting that the mentioned strains are the most common etiologic agents causing osteomyelitis. Another approach is the synthesis of composites based on HA and polymers loaded with drugs. Benedini *et al.*(14) proposed composites containing HA and sodium alginate loaded with ciprofloxacin. It is also possible to obtain the synergistic effect of antibiotics loaded into the biomaterial. Suchý *et al.*(15) proposed a novel electrospun composite based on collagen and HA loaded with vancomycin, gentamicin, and their combination. The antibacterial properties of HA have also been developed by combinations with biopolymers such as agarose and graphene oxide.^16^

It is worth noting that besides biopolymers, there is a variety of synthetic polymers that exhibit antimicrobial properties, *e.g.*, metal–organic frameworks (MOFs). These porous coordination polymers have numerous advantages over typical porous materials, including versatile composition and micro-/nanoscale structures, large specific surface area, or high porosity.^17^ Since the structural properties of MOFs may be freely adapted, they can be applied in a variety of fields, including gas storage and separation,^18^ water purification,^19^ drug delivery,^20,21^ or catalysis.^22,23^ The antimicrobial activity of MOFs can be a result of drug incorporation in the particle voids or properties of the metal component in the framework.^24^ The great advantage of MOFs over traditional antibacterial agents is the low possibility of resistance development due to the multiple mechanisms of antibacterial action.^25^ One of the most popular MOFs used as a component for antibacterial composites is Cu-BTC, also known as Cu-HKUST-1. The silk fibers containing Cu-BTC proposed by Abbasi *et al.*(26) exhibited high antibacterial activity against *E. coli* and *S. aureus.* Antibacterial activity against *E. coli* also shows a composite based on Cu-BTC and cellulosic fibers. What is more, there are also Cu-HKUST-1-based materials with polyvinyl alcohol^27^ and polyester^28^ that showed great activity against selected bacteria strains.

Taking into account the presented background, in this work, we demonstrated a novel composite based on HA nanoparticles and Cu-HKUST-1 to act as a biocompatible material (Scheme 1). The enhancement of the bioapplication of the composite over pristine HA was achieved by the confirmed antimicrobial properties and increased nanomechanical properties. Noteworthily, Cu-HKUST-1 was selected among four Cu-based MOFs as the most promising antimicrobial agent (Table 1). The minimal inhibitory concentration (MIC) values of Cu-HKUST-1 against microorganisms tested were in the range of 2–4 mg/mL, while the minimal biocidal concentration (MBC) values of this compound were equal to 3 mg/mL against all tested bacteria and 4 mg/mL against *Candida albicans*.

**Scheme 1:**
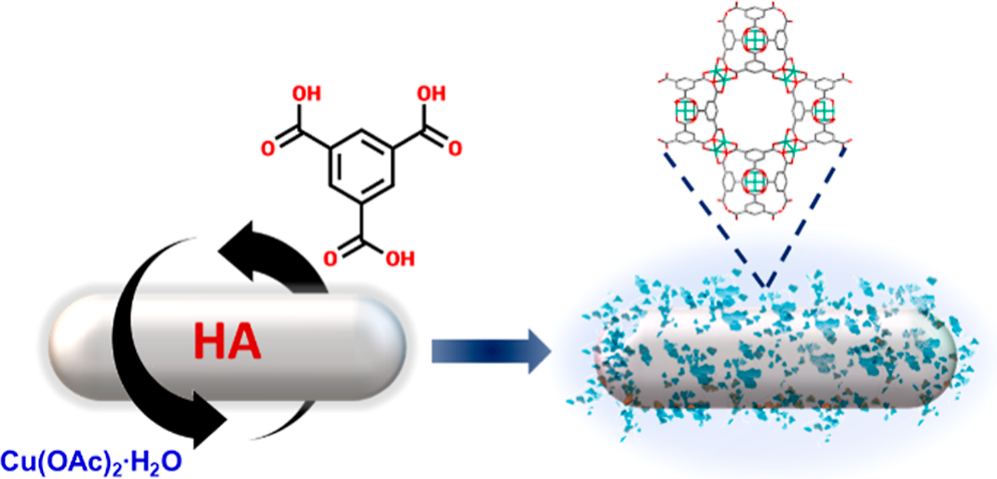
Preparation of the Composite Material Cu-HKUST-1@HA Following a Layer-by-layer Method

**Table 1:**
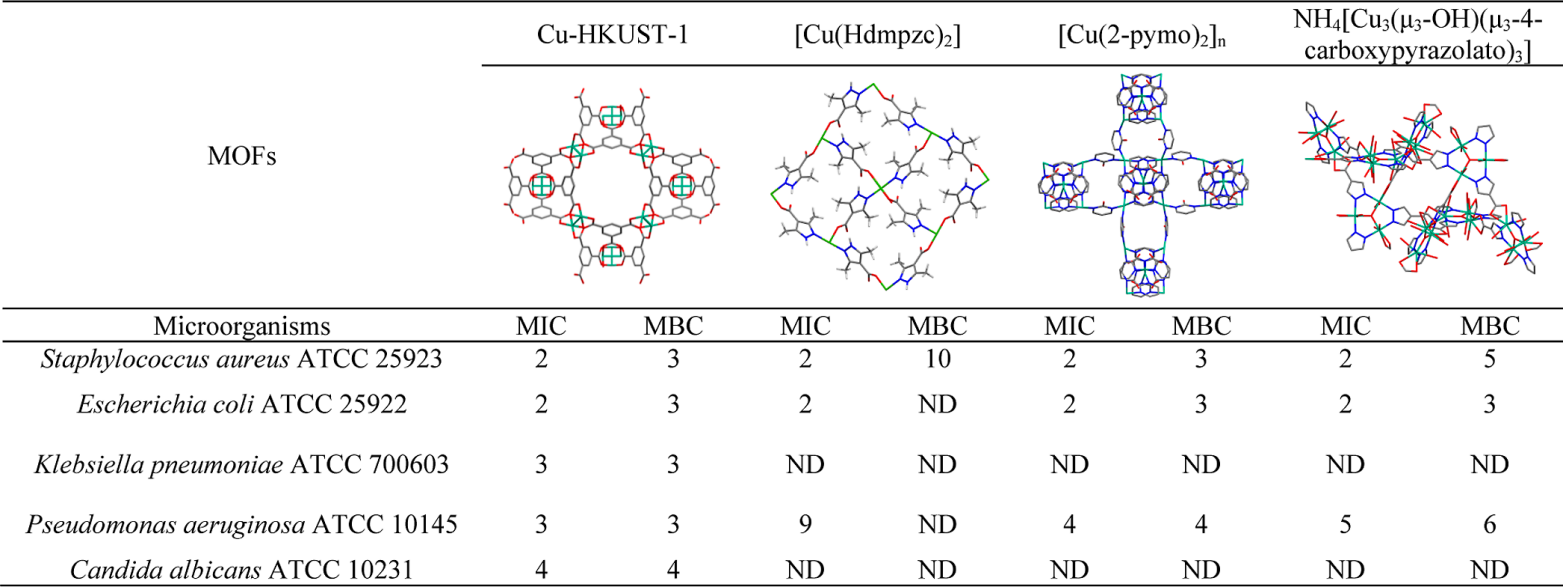
Minimal Inhibitory Concentrations and Minimal Biocidal Concentrations (mg/mL)^a^

a ND = not determined in the tested concentration range (1–10 mg/mL).

The preparation of Cu-HKUST-1,^29^ [Cu(Hdmpzc)_2_] (H_2_dmpzc = 3,5-dimethyl-1*H*-pyrazol-4-carboxylic acid),^30^ [Cu(2-pymo)_2_]_*n*_ (Hpymo = 2-hydroxypyrimidine),^31^ and NH_4_[Cu_3_(μ_3_-OH)(μ_3_-4-carboxypyrazolato)_3_]^32^ was carried out according to a previously reported method (for more details see the Supporting Information). The synthesized MOFs were systematically characterized by powder X-ray diffraction (PXRD) and infrared spectroscopy (IR), confirming the purity of the isolated material (see Supporting Information Figures S1–S4). Rod-shaped HA nanoparticles, Ca_10_(PO_4_)_6_(OH)_2_, were synthesized by the wet precipitation method, wherein phosphoric acid was slowly added into a calcium hydroxide solution. Afterward, a post-synthetic hydrothermal treatment was carried out, which reduced HA agglomeration and improved particle morphology.^33^ HA nanorods (71 ± 18 nm long and 27 ± 4 nm wide) (Figure S5) revealed the Ca/P molar ratio equal to 1.50 (based on ICP-OES).

Regarding the new composite material Cu-HKUST-1@HA, it was synthesized following a layer-by-layer method (Scheme 1). This strategy was also described by Abbasi *et al.*(26) for silk fibers coated with Cu-HKUST-1 under ultrasound irradiation. According to our protocol, HA nanoparticles were suspended in an ethanolic solution containing 1,3,5-benzenetricarboxylic acid (H_3_BTC) and kept at room temperature for 1 h. Afterward, the nanoparticles were recovered by centrifugation, washed with fresh ethanol, and resuspended at RT for 1 h in an ethanolic solution of copper(II) acetate hydrate. The obtained composite nanoparticles were washed and centrifuged prior to the following cycle. This procedure was repeated 10 times. The growth of Cu-HKUST-1 on HA nanoparticles was first confirmed by PXRD studies. The presence of the most intense reflections of Cu-HKUST-1 at 2θ = 9.3, 11.7, and 13.2° next to those characteristic for HA indicates the dual nature of the new composite (Figure 1a). Additionally, IR spectroscopy confirmed the successful deposition of Cu-HKUST-1 on HA nanoparticles’ surface (Figure 1b). The characteristic bands of the BTC ligand (1646–1376 cm^–1^ region attributed to C=O and C=C stretching vibrations and two bands at 764 and 731 cm^–1^ associated to C–H bending vibrations)^27^ as well as a less intense band at 491 cm^–1^ corresponding to the Cu–O bond^27^ were observed in the Cu-HKUST-1@HA spectrum. Likewise, the Raman spectrum of the new composite (Figure S6) revealed both main bands characteristic for PO_4_^3–^ vibrations of HA (432, 590, 963, and 1046 cm^–1^)^34^ as well as low intense bands below 600 cm^–1^ due to the copper ions in the Cu-HKUST-1 framework and bands in the range of 700–1800 cm^–1^ as a result of vibrational modes of the BTC ligand.^35^ Among them are bands at 1615 and 1006 cm^–1^, characteristic of C=C modes of the benzene ring, 745 and 829 cm^–1^ designated as C–H bending vibrations, and the bands due to C–O–O symmetric and C–O–O asymmetric units centered at 1465 and 1553 cm^–1^.^35^ The spectroscopic characterization of Cu-HKUST-1@HA was extended to include electron paramagnetic resonance (EPR) studies. The EPR spectra of a solid state at two temperatures 273 and 77 K were also registered for pristine Cu-HKUST-1 as a reference. MOFs synthesized in two different manners: solvothermal (Cu-HKUST-1_solv) and mechanochemical under a LAG condition (Cu-HKUST-1_mech) were analyzed to find out the differences in the Cu-HKUST-1 structure due to the synthesis method. On the other hand, it was interesting to correlate these results to the MOF structure in the composite prepared by the layer-by-layer method. At room temperature (Figure S7), a broad and weak EPR spectrum was observed for all samples with *g* ≈ 2.09–2.14, which can be attributed to spin exchange between the Cu^2+^ paddlewheels *via* the linkers, resulting in averaging of the anisotropic spectral features from individual Cu dimers.^36^ Upon lowering the temperature to 77 K, the EPR spectrum of Cu-HKUST@HA started exhibiting resolved resonance lines that are not typical for randomly oriented triplet states, which are attributed to Cu^2+^–Cu^2+^ dimers. In contrast, it shows traces of a hyperfine structure, which occurs in the case of equivalent copper ions (nuclear spin *I* = 3/2) or monomers (Figure 1c). The parameters obtained from the simulation were *g*_*xx*_ = 2.08, *g*_*yy*_ = 2.13, *g*_*zz*_ = 2.36; *A*_*xx*_ = *A*_*yy*_ = 20 G, *A*_*zz*_ = 130 G (Figure 1d). In our case, such an effect was not obvious because the starting compound was a dimer. In the Cu-HKUST-1_solv, obtained by the typical method, the signals were observed in zero field splitting and about 6000 G. For comparison in the mechanochemically synthesized material, only a peak centered at 3200 G was noticed, which is assigned to uncoupled Cu^2+^ (Figure 1d). This suggests that the mechanochemical synthesis method results in a different electronic structure compared to the solvothermal synthesis. Based on these results, it was suggested that both the monomeric and dimeric forms of copper contribute to the Cu-HKUST-1@HA structure.

**Figure 1:**
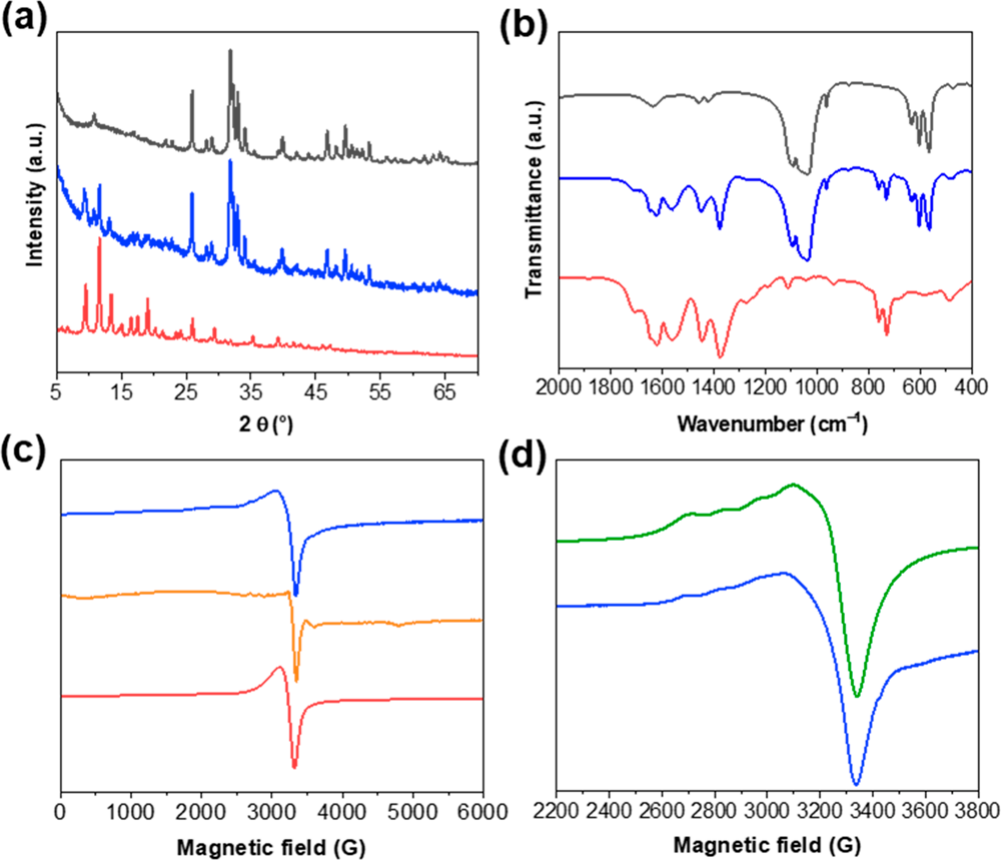
PXRD patterns (a) and IR spectra (b) of HA (black), Cu-HKUST-1@HA (blue), and Cu-HKUST-1 (red). For the composite, the presence of characteristic reflections or bands of both HA and Cu-HKUST-1 is observed. EPR spectra (c,d) of Cu-HKUST-1@HA (blue), Cu-HKUST_solv (orange), Cu-HKUST_mech (red), and simulation for Cu-HKUST-1@HA (green).

Transmission electron microscopy (TEM) images demonstrated that Cu-HKUST-1@HA maintains the rod-shaped morphology of hydrothermally treated HA nanoparticles with a colloidal layer of Cu-HKUST-1 (Figure 2). Moreover, high-resolution transmission electron microscopy (HR-TEM) and fast Fourier transform analysis (Figure 2c,d) allowed the identification of crystalline phases in the composite. Surprisingly, in addition to the following lattice planes characteristic of HA (130), (002), (211), (112), (300), the Cu_2_O phase with lattice plane (200) was found. The fine Cu_2_O crystallites observed in TEM images show very good dispersion. So far, Siddiqui *et al.* have also observed Cu_2_O formation during Cu-HKUST-1 synthesis.^35^ The presence of different Cu species was further confirmed by XPS studies (Figure S8). Indeed, the deconvolution of the Cu 2p_3/2_ peak revealed two chemical states of copper. The peak located at around 932.56 eV was attributed to the presence of Cu_2_O.^37,38^ Analysis of the percentages of individual Cu components indicated a 5.12% content of this Cu(I) species. Besides, peaks of Ca and P due to HA with binding energies of 351.5 eV (Ca 2p_1/2_), 347.9 eV (Ca 2p_3/2_), 135.2 eV (P 2p_1/2_), and 133.4 eV (P 2p_3/2_) were observed (Figures S9 and S10). Textural characterization of the new composite revealed that Cu-HKUST-1 coating increased the BET surface area from 40 m^2^ g^–1^, for HA nanoparticles, to 140 m^2^ g^–1^ for Cu-HKUST-1@HA (Figure S11). In addition, the N_2_ sorption isotherm is a distinct combination of type I and IV isotherms; hence, the synthesized materials have micro- and mesopores.^39^ Further characterization with the use of TGA studies (Figure S12) indicated high thermal stability of Cu-HKUST-1@HA similar to that for HA. Although the profile of TGA curves corresponds to pristine Cu-HKUST-1, the low weight loss (up to 0.4%) indicates a low MOF layer thickness in the composite. Neglecting the presence of Cu_2_O, the chemical composition of the composite was calculated as 0.04 mol Cu-HKUST-1@1 mol HA based on ICP-OES and TGA studies (see Supporting Information Figure S12).

**Figure 2:**
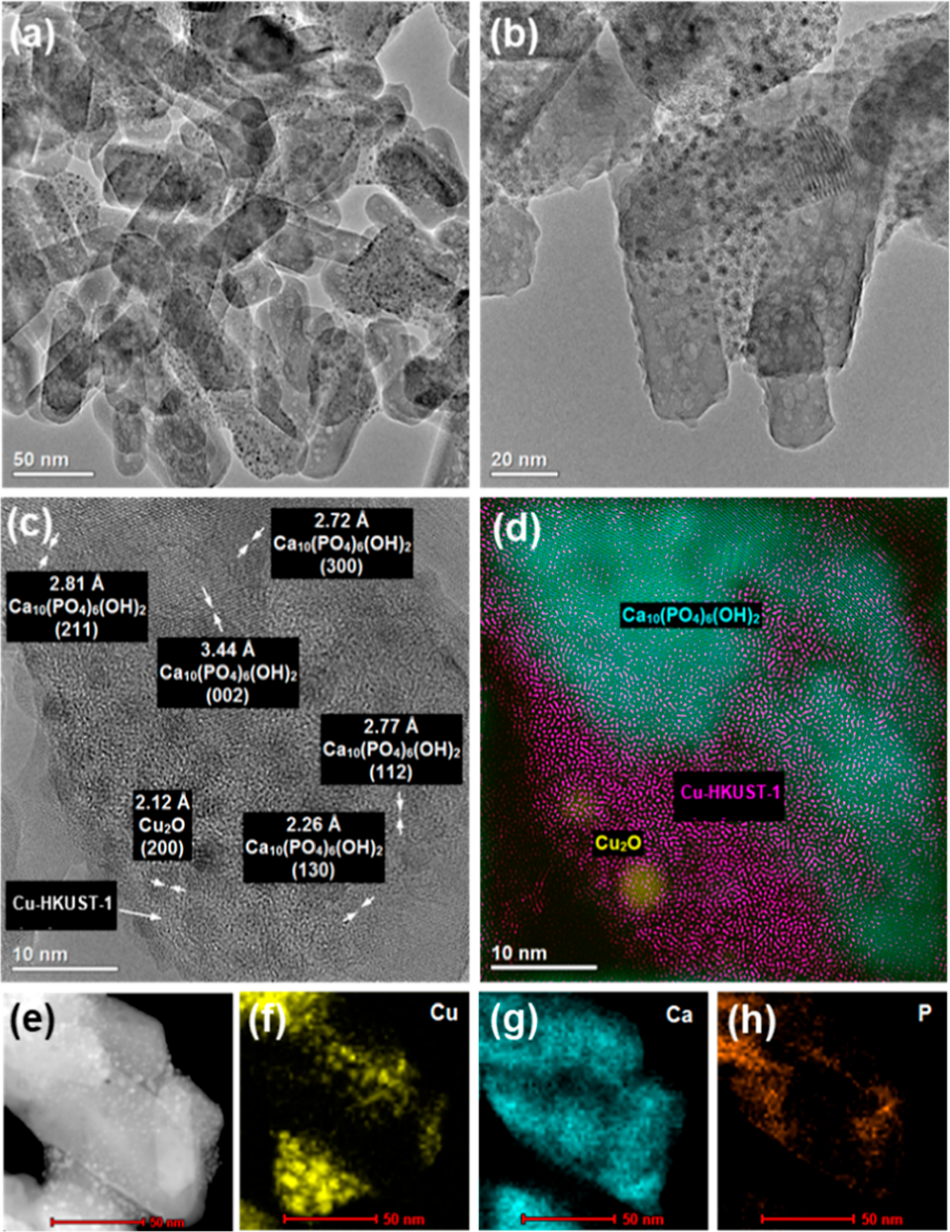
HR-TEM images of Cu-HKUST-1@HA (a,b), with phase analysis (c,d) and EDS elemental mapping (e–h) showing elongated crystallites of HA coated with Cu-HKUST-1.

Once the new composite was structurally, spectroscopically, and texturally characterized, we have studied properties important from a bioapplication viewpoint. First, nanomechanical properties were considered. The shape of the hysteresis curve for tested materials confirmed elastic–plastic deformation mode active during indentation (Figure 3a).^40^ A significant decrease in indentation depth was noted for Cu-HKUST-1@HA. As observed, the presence of the MOF layer in the composite resulted in a significant increase in hardness and Young’s modulus compared to pristine HA (Table 2). Besides, HA after Cu-HKUST-1 coated brought out an elastic energy increase of about 24.76%, while the plastic/total energy ratio (ductility index, D) decreased by *ca.* 18.27%. For implant materials, the mechanical properties of biomaterials should approximate the mechanical properties of the tissues surrounding the implant. In the case of the tested materials, it is a bone tissue whose mechanical behavior should be a reference. Importantly, in the case of our composite Young’s Modulus equal to that of human cortical bone, 10–30 GPa,^41^ was obtained. The increase in nanohardness and Young’s modulus of the Cu-HKUST-1@HA powder can be attributed to the consolidation mechanisms present in the material in the matrix. Similar correlations were obtained for the Mg–SiC powder composites.^42^ The mechanical properties, such as the elastic strain to failure of the materials (H/E) and the resistance of a material to plastic deformation (H^3^/E^2^), were also found using the nanoindentation technique.^43,44^ The H/E ratio characterizes the resistance of the material to elastic deformation and H^3^/E^2^ ratio allows to estimate the material’s ability to dissipate energy at plastic deformation during loading.^45^ Both ratios may characterize approximately the wear resistance of a material, either in bulk or in coating form. In the case of the tested materials, a notable improvement of both coefficients was obtained for the new composite. Since the materials intended for implants are subjected to continuous loading, the increase of the determined coefficients supports the idea of implantation of Cu-HKUST-1@HA instead of HA only.

**Figure 3:**
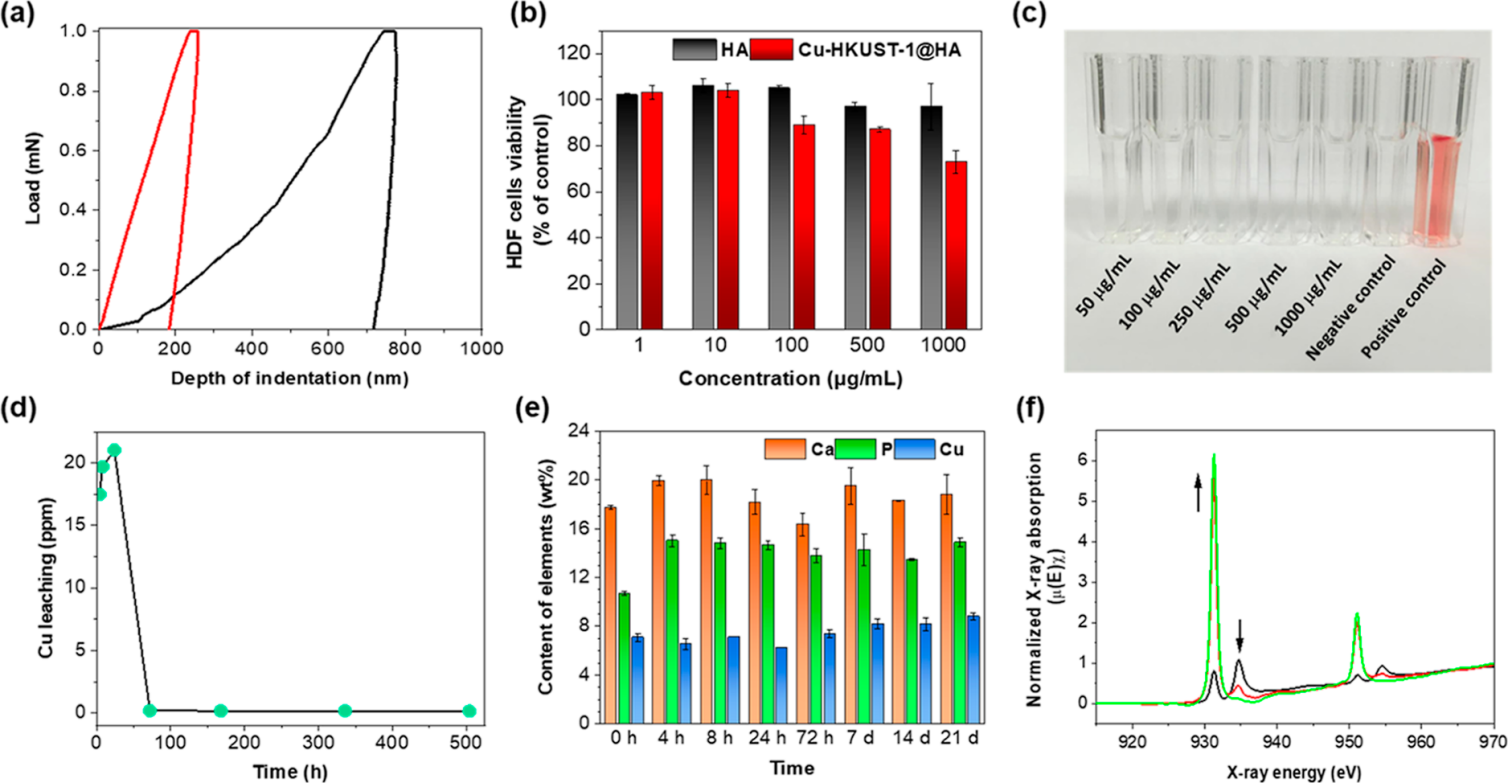
(a) Single hysteresis loops for Cu-HKUST-1@HA (red) and HA (black). (b) Viability of HDFs growing in the presence of HA and Cu-HKUST-1@HA for 24 h. (c) Hemolysis study results of test samples at different Cu-HKUST-1@HA concentrations. (d) Cu leaching from Cu-HKUST-1@HA during the incubation in DPBS (37 °C, 4 h to 21 days) monitored by ICP-OES. (e) Analysis of the composition of Cu-HKUST-1@HA during the incubation in DPBS monitored by ICP-OES. (f) Normalized Cu L-edge absorption spectra of Cu-HKUST-1@HA treated in DPBS for 0 h (black), 8 h (red), and 72 h (green).

**Table 2:** Nanoindentation Results^a^

specimen	hardness (GPa)	Young’s modulus (GPa)	H/E ratio (−)	H^3^/E^2^ ratio (Pa)	elastic energy (nJ)	plastic/total energy ratio, D (−)
HA	0.0705 ± 0.0118	5.69 ± 1.46	0.0127 ± 0.0014	11.10 ± 0.92	0.0240 ± 0.0033	0.9250 ± 0.0286
Cu-HKUST-1@HA	0.7469 ± 0.1124*	13.76 ± 3.12*	0.0550 ± 0.0042*	2230.73 ± 84.77*	0.0319 ± 0.0063*	0.7560 ± 0.0196*

a *n* = 5, *significantly different results compared to the HA specimen, according to one-way ANOVA followed by Bonferroni’s multiple comparison test, *p* < 0.05.

Further research on the new composite revealed that the zeta potential of the Cu-HKUST-1@HA remains negative, equal to −16.3 mV, in Dulbecco’s phosphate-buffered saline (DPBS) buffer (pH 7.4) (−14.6 mV for HA), which is expected to have a significant favorable effect on the attachment and proliferation of bone cells.^46^ When considering biocompatibility, the *in vitro* cell viability in the presence of a new composite was determined using human dermal fibroblasts (HDFs) as a model cell line. Importantly, no considerable changes in the HDF cell viability were observed after 24 h (Figure 3b) of incubation with Cu-HKUST-1@HA in a concentration range of 1–1000 μg/mL. We have also tested the HDF cells’ viability in higher concentrations of Cu-HKUST-1@HA, and this allowed us to calculate the EC_50_ value as equal to 5 mg/mL. Remarkably, the material did not cause hemolysis in the range of concentrations tested (up to 1000 μg/mL). After the exposure to Cu-HKUST-1@HA, the determined level of hemolysis in all samples was on the level of negative control (erythrocytes in PBS), which confirms the excellent hemocompatibility of this material (Figure 3c). This is crucial because, to date, copper has been widely used in bone tissue engineering due to its excellent properties; however, it suffers from high toxicity.^47^ Therefore, reducing the toxicity of copper-based biomaterials remains a great challenge. Slow release of Cu^2+^ in sufficient quantity to induce an antibacterial effect may be a strategy in bone tissue regeneration. ICP-OES studies of the filtrate after different incubation times (37 °C, in DPBS, a buffer simulating physiological fluid) clearly revealed Cu release within 24 h. At this point, the composite reduced the Cu content by *ca.* 0.84 wt %. Afterward, subsequent Cu incorporation into the studied material was noted (Figure 3d). On the other hand, PXRD (Figure S13a) and IR (Figure S14) studies confirmed MOF layer degradation in Cu-HKUST-1@HA under physiological conditions (37 °C, pH = 7.4) in less than 4 h. However, analysis of the composition of the material after different incubation times clearly indicated an increase in P (*ca.* 4% wt during 21 d) with no loss of Cu content (Figure 3e). In addition, energy-dispersive spectrometry (EDS) maps of the analyzed area showed a large dispersion of Ca and P localized in the same area, indicating the formation of calcium phosphate due to MOF degradation and further interaction with the buffer (Figure S15). Consequently, on the IR spectra, a new band at 993 cm^–1^ appeared after incubation (Figure S14) that is attributed to P–O vibrations of PO_4_^3–^.^48^ It correlates with the PXRD results, and more specifically, new reflections observed at 8.9 and 12.8° (less intensity), indicating the formation of Cu_3_(PO)_4_·3H_2_O^49^ (Figure S13b).

To study more in-depth the structure of Cu-HKUST-1@HA and structural changes induced under conditions that simulate body fluid, X-ray absorption spectroscopy (XAS) spectra were obtained on the selected 4s and 3d metals (Ca, Cu) L-edge and the O K-edge (Figure S16). The normalized Cu L-edge X-ray absorption spectrum of pristine Cu-HKUST-1 and Cu-HKUST-1@HA before or after incubation in DPBS for selected times (8 and 24 h and 21 days, Figures S17, 3f) involved the Cu 2p_3/2_ → 3d and 2p_1/2_ → 3d transitions and consists of two major peaks that split ∼20 eV (3/2 × the 2p core spin–orbit coupling), with an intensity ratio of ∼2:1, where *J* = 3/2 and 1/2, correspond to the L_3_-edge (∼930 eV) and the L_2_-edge (∼950 eV), respectively.^50^ In the measured Cu XAS spectra, additionally, apart from two main regions attributed to the dipolar excitations of 2p_3/2_ electron (L_3_-edge) and 2p_1/2_ electron (L_2_-edge) into unoccupied Cu 3d states, two additional transitions are observed (∼934 and ∼954 eV) for *J* = 3/2 and 1/2, respectively. It is worth noting that these transitions are better described as a shakeup transition [2p^6^3d^9^4s^0^L^0^] → [2p^5^3d^10^4s^1^L^+^], where the internal electronic transitions of the metal are accompanied by a ligand to metal electron transfer LMCT,^51−54^ L^0^ and L^+^ representing the neutral and charged ligands. The Cu XAS splitting at the Cu-HKUST-1 spectra (Figure S17a) was also observed for copper dimers, which is very common in copper chemistry, with oxygen donor atoms surrounding each metal center or for cuprates with the tetragonal pyramidal geometry and D_4h_ geometry, respectively.^55,56^ This conclusion might be supported because the O K-edge XAS spectrum of Cu-HKUST-1 consists of a pronounced pre-peak around 527.6 eV (Figure S18a, red line), suggesting a significant ligand-hole character of its ground state.^57^ The O K-edge pre-peak is less intense for HA and Cu-HKUST-1@HA (Figure S18b,c). In the case of Cu-HKUST-1@HA (Figure S17b), these dipole-allowed post-edge peaks corresponding to 2p → d^10^4s^1^L^+^ transitions were more intensive than the original in Cu-HKUST-1 (Figure S17a), suggesting a more distorted local structure of central atoms and mixed electron state and more significant 3d-4s orbital mixing attributed to the covalency of the ligand–metal bonds as well as a weak 2p-3d orbital mixing between the 2p ligand (carboxylate and phosphate) and 3d metal orbitals attributed to π bonding interactions. The intensity of the Cu L-edge satellite peaks decreased with increasing intensity of the Cu L-edge main peaks when the composite was treated with DPBS buffer up to 72 h (Figure 3f) when the Cu local structure had been changed on a HA surface. After 21 d, the satellite peaks at ∼934 and ∼954 eV were slightly rebuilt (Figure S19). In the present case, the XAS result has shown that MOF layer degradation causes a decrease of the ionicity of ligand–copper bonds and a blue shift of the free d level. The Ca L-edge XAS spectra did not change significantly, and no energy shift of features was observed during treatment of the composite with buffer (Figure S20), suggesting a lack of influence of calcium ion in bonding with Cu-HKUST-1 with respect to the hydrated copper(II) phosphate formation after MOF degradation. The intensity of satellite pre-peaks and main 2p-4s transition peaks (∼349 and ∼352 eV) decreased only up to 72 h and, and after 21 d, it slightly increased.

It should be concluded that the XAS spectra correctly characterize changes in the covalency of copper–oxygen bonds and changes in the local structure of copper in the MOF component of Cu-HKUST-1@HA treated in buffered aqueous solutions. Moreover, the composite undergoes gradual transformation along with the degradation of the MOF structure but still retains the layered structure of the original material.

Once the new composite was fully characterized, we studied the antimicrobial activity of Cu-HKUST-1@HA. As far as we know, this is the first report of a biomaterial/MOF interface of antibacterial composites dedicated to bone tissue regeneration. The results showed that HA tested individually was not active against microorganisms in the tested concentration range, while their coating with Cu-HKUST-1 affected the antibacterial activities of the composite (Table 3). However, it was noted that the thin MOF layer provided the lower antimicrobial activity of the composite compared to pristine Cu-HKUST-1. Cu-HKUST-1@HA was most active against Gram-positive (*S. aureus*) than Gram-negative bacteria (*E. coli*, *K. pneumoniae* and*P. aeruginosa*). The MIC value of the new composite against *S. aureus* was found to be 2 mg/mL (likewise for Cu-HKUST-1), while the MBC was 5 mg/mL (Table 3). The MIC and MBC values against Gram-negative bacteria ranged from 4 to 9 and 6 to 9 mg/mL, respectively. Similarly, the higher antibacterial properties against Gram-positive *S. aureus* were reported by Ren *et al.*(58) for the HKUST-1/chitosan film. The difference in the antimicrobial activity of these materials was ascribed to the presence of a thick outer membrane of cell walls in Gram-negative bacteria that may prevent MOFs from penetrating into bacterial cells and the different isoelectric points of the envelope of these two types of bacteria.^59^ The Gram-positive bacteria have the isoelectric point of the cell wall of around 4–5 and produce a more negatively charged surface than the Gram-negative bacteria, which have the isoelectric point of the surface of around 2–3 at the same pH value.^58^ In turn, the thick cell wall of eukaryotic*C. albicans* cells, composed of glucans, chitin, and glycoproteins^60^ may also highly prevent diffusion of the tested compound into cells; therefore, the antifungal activity of Cu-HKUST-1@HA was not observed in the tested concentration range.

**Table 3:** MICs and MBCs (mg/mL)

bacterial strains	HA		Cu-HKUST-1@HA		Cu-HKUST-1	
	MIC	MBC	MIC	MBC	MIC	MBC
*Staphylococcus aureus* ATCC 25923	>10	>10	2	5	2	3
*Escherichia coli* ATCC 25922	>10	>10	4	6	2	3
*Klebsiella pneumoniae* ATCC 700603	>10	>10	9	9	3	3
*Pseudomonas aeruginosa* ATCC 10145	>10	>10	6	6	3	3
*Candida albicans* ATCC 10231	>10	>10	>10	>10	4	4

It is also worth mentioning that from an application point of view, the antibacterial activity of Cu-HKUST-1@HA against*S. aureus*is of importance. The reason is that this strain is commonly found in wound infections and can also be implicated in osteomyelitis.^61^

In conclusion, a thin layer of Cu-HKUST-1 coating on HA has given this biomaterial new therapeutic qualities, namely, antibacterial properties. Noteworthily, a marked improvement in the nanomechanical properties of the composite was achieved while maintaining bio- and hemocompatibility. Improved properties of pristine HA and antibacterial effect determine Cu-HKUST-1@HA as a new candidate for the components of multifunctional implants.

## Supporting Information Available

General methods; synthesis of materials; details of X-ray absorption studies and other measurements to determine the nanomechanical properties; buffer stability test; *in vitro* cytotoxicity assay; hemolytic and antimicrobial activity; IR, Raman, EPR, XPS, and XAS spectra; TEM images; X-ray powder diffractograms; N_2_ sorption isotherms; TGA; and ICP-OES (PDF)
