# Supplementary material for: Cu-HKUST-1 and Hydroxyapatite–The Interface of Two Worlds toward the Design of Functional Materials Dedicated to Bone Tissue Regeneration
Source: ACS Biomater Sci Eng. 2023 Aug 1;9(8):4646–53. doi: 10.1021/acsbiomaterials.3c00594 (PMC10428089; doi:10.1021/acsbiomaterials.3c00594)
Supplement: Supplementary file 1 — ab3c00594_si_001.pdf [file ab3c00594_si_001.pdf]

Supporting Information

Cu-HKUST-1 and hydroxyapatite - the interface of two worlds towards the design of functional materials dedicated to bone tissue regeneration

Marzena Fandzloch,[†,*] Weronika Bodylska,[†] Joanna Trzcińska-Wencel,[‖] Patrycja Golińska,[‖] Katarzyna Roszek,[‖] Joanna Wiśniewska,[⁑] Michał Bartmański[‡], Agnieszka Lewińska,[§] Anna Jaromin[§§]

[†]Institute of Low Temperature and Structure Research, Polish Academy of Sciences, Okólna 2, 50-422 Wrocław, Poland

[‖]Faculty of Biological and Veterinary Sciences, Nicolaus Copernicus University in Toruń, Lwowska 1, 87-100 Toruń, Poland

[⁑]Faculty of Chemistry, Nicolaus Copernicus University in Toruń, Gagarina 7, 87-100 Toruń, Poland

[‡]Faculty of Mechanical Engineering and Ship Technology, Gdańsk University of Technology, Gabriela Narutowicza 11/12, 80-233 Gdańsk, Poland

[§]Faculty of Chemistry, University of Wrocław, F. Joliot-Curie 14, 50-383 Wrocław, Poland

[§§]Department of Lipids and Liposomes, Faculty of Biotechnology, University of Wrocław, F. Joliot-Curie 14a, 50-383 Wrocław, Poland

Index

1. General methods **S2**
2. Synthesis of the materials **S3**
3. X-ray absorption studies **S5**
4. Nanomechanical properties **S5**
5. Buffer stability test **S6**
6. *In vitro* cytotoxicity assay **S6**
7. Hemolytic activity **S6**
8. Antimicrobial activity **S7**
9. Additional figures **S8**
10. Bibliography **S24**

1. General methods

All solid products were analyzed by powder X-ray diffraction (PXRD) using a X'Pert PRO diffractometer (PANalytical) with Cu Kα radiation (λ = 1.5406 Å). The mid-infrared spectra (IR) were measured using the Nicolet iSTM50 FT-IR spectrometer. The spectra were recorded for the KBr pellets. The micro-Raman apparatus (inVia™ Renishaw) was used to register the Raman spectra with a 514 nm excitation line. Thermogravimetric analysis (TGA) was performed using a Setaram SETSYS TG-DTA 16/18 at a heating rate of 10 ºC/min in flowing air. The specific surface area was determined based on N_2 sorption measurements. The N_2 adsorption-desorption isotherms were obtained at 77 K using a Micromeritics 3Flex Surface Characterization Analyzer. Prior to isotherm acquisition, the materials were activated and outgassed (150 ºC, 1.3 kPa) for 12 h. MicroActive software was used to determine specific surface area according to the Brunauer–Emmett–Teller (BET) method. HR-TEM images with Fast Fourier Transform (FFT) and STEM-EDS elemental maps were performed using a FEI Titan G2 60-300. The particle size of HA was calculated from the size of 100 particles determined by ES Vision software using TEM images. The powders and filtrates composition were characterized using iCAP™ 7400 ICP-OES Analyzer. To quantify the content of Ca, P and Cu in HA and Cu-HKUST-1@HA by ICP-OES, 1-2 mg of powders were degraded in 3 mL of HNO_3. Afterwards, the samples were diluted with distilled water up to 23 mL. On the other hand, the filtrates after a defined time of Cu-HKUST-1@HA incubation in DPBS (4 h, 8 h, 24 h, 72 h, 7 d, 14 d, and 21 d), were acidified with HCl (5 mL) prior to analysis of Cu leaching. Zeta potentials were measured with Zetasizer Nano ZS (Malvern Instruments) for HA and Cu-HKUST-1@HA (1.5 mg/mL in DPBS or distilled water) sonicated for 5 min before measurement. The potential was measured six times each in two independent experiments (each measurement being the average of 100 runs) both in DPBS and H_2O. The mean values and standard deviations were equal to -16.3 ± 1.7 mV for Cu-HKUST-1@HA, -14.6 ± 3.7 mV for HA in DPBS, and -9.1 ± 0.7 mV for Cu-HKUST-1@HA and -14.2 ± 2.3 mV for HA in H_2O. The EPR spectra were measured at 77 K using a Bruker Elexsys 500 CW-EPR spectrometer operating at the X-band frequency (~9.7 GHz), equipped with frequency counter (E 41 FC) and NMR teslameter (ER 036TM). The spectra were measured with a modulation frequency of 100 kHz, microwave power of 10 mW, modulation amplitude of 10 G, time constant of 40 ms and a conversion time of 160 ms. The first derivative of the absorption power was recorded as a function of the magnetic field value. The experimental spectra were simulated using the computer program DoubletExact (S = 1/2), written by Prof. Andrew Ozarowski from NHMFL, Florida State University. The Cu-HKUST-1 used was obtained in two different manners: solvothermal (Cu-HKUST-1_solv) according to known procedure[1] and mechanochemical (Cu-HKUST-1_mech) described in section 2.1.

2. Synthesis of the materials

Copper(II) acetate monohydrate (≥98%), phosphoric acid (85%), phosphate-buffered saline, Dulbecco's formula (DPBS, 10X), and 1,3,5-benzenetricarboxylic acid (H_3btc) (98%) were purchased from Alfa Aesar. Calcium hydroxide (≥96%), copper(II) nitrate trihydrate (99-104%), 3,5-dimethyl-1*H*-pyrazol-4-carboxylic acid (H_2dmpzc), 2-hydroxypyrimidine hydrochloride (98%) was purchased from Sigma-Aldrich. 1H-Pyrazole-4-carboxylic acid (97%) was purchased from Thermo Fisher Scientific. Ethanol (99.8%), nitric acid (65%), hydrochloric acid (35-39%), acetone, and ammonia hydroxide (25%) were purchased from Avantor Performance Materials Poland S.A. All chemical reagents were used as received without further purification. Ultra-pure water (18.2 mΩ cm^{-1}) was used throughout.

2.1. Synthesis of Cu-HKUST-1

Cu-HKUST-1 was prepared by a liquid-assisted grinding (LAG) method previously described by Steenhaut et al. with some modifications.[2] Typically, $Cu(OAc)_2 \cdot H_2O$ (0.040 g, 0.2 mmol) and H_3btc (0.028 g, 0.13 mmol) were pre-ground in an agate mortar and then introduced into a milling tube together with 0.5 mL of ethanol. The mixture was subjected to milling at 6000 rpm for 30 min in the homogenizer (IKA ULTRA-TURRAX® Tube Drive Disperser). Milling was carried out in an appropriate tube of 15 mL containing ten stainless-steel balls of 5 mm. Afterwards, the resulting precipitate was washed twice with ethanol (99.8%) (2 × 10 mL) and dried in vacuo.

2.2. Synthesis of [Cu(Hdmpzc)$_2$]

[Cu(Hdmpzc)$_2$] was synthesized according to a previously published method.[3] Briefly, H_2dmpzc (0.070 g, 0.5 mmol) and $Cu(OAc)_2 \cdot H_2O$ (0.100 g, 0.5 mmol) were added to distilled water (10 mL). The mixture was left overnight at reflux under stirring. The formation of a brown suspension was observed, while a violet precipitate was finally formed after cooling to room temperature. The resulting precipitate was filtered off, washed with distilled water (2 × 10 mL), and dried in vacuo.

2.3. Synthesis of [Cu(2-pymo)$_2$]$_n$

[Cu(2-pymo)$_2$]$_n$ was synthesized according to a previously published method.[4] Typically, an aqueous ammonia solution (NH_4OH/H_2O = 2:8, 30 mL) containing $Cu(NO_3)_2 \cdot 3H_2O$ (0.242 g, 1 mmol) and 2-hydroxypyrimidine·HCl (0.265 g, 2 mmol) gives a dark purple solid after leaving the solution in open air for 1 day. The resulting precipitate was filtered off, washed with distilled water (2 × 10 mL), acetone (1 × 5 mL), and dried in vacuo.

2.4. Synthesis of $NH_4[Cu_3(\mu_3\text{-}OH)(\mu_3\text{-}4\text{-carboxypyrazolato})_3]$

$NH_4[Cu_3(\mu_3\text{-}OH)(\mu_3\text{-}4\text{-carboxypyrazolato})_3]$ was prepared according to the synthesis previously described by the Navarro group.[5] For this purpose, 1H-pyrazole-4-carboxylic acid (0.224 g, 2 mmol) and $Cu(NO_3)_2 \cdot 3H_2O$ (0.483 g, 2 mmol) were dissolved in an aqueous ammonia solution (NH_4OH/H_2O = 1:15, 30 mL) and left for 3 days while dark blue crystals were formed. Afterward, these crystals were centrifuged, washed with distilled water (2 × 20 mL) and ethanol (96%) (1 × 20 mL), and dried for 24 h at 50 ºC.

2.5. Synthesis of hydroxyapatite (HA)

HA nanoparticles ($Ca_{10}(PO_4)_6(OH)_2$) with rod-like shapes were synthesized according to a previous method.[6] Briefly, H_3PO_4 (0.15 M) was slowly dropped to an aqueous suspension of $Ca(OH)_2$ (ca. 0.17 M), resulting in the precipitation of HA. The reaction mixture was further stirred at 37 °C for 24 h and left standing for the next 24 h. Afterwards, the precipitate was separated by centrifugation, washed with water (3 × 20 mL), and dried at 60 ºC overnight. Then, to obtain the rod-like shape, the HA powder was hydrothermally treated (at 200 ºC for 24 h). After cooling to room temperature, the HA powder was washed with distilled water (2 × 20 mL) and dried at 60 ºC overnight.

ICP-OES: Ca/P molar ratio = 1.50.

2.6. Synthesis of Cu-HKUST-1@HA

The new Cu-HKUST-1@HA composite was prepared following a layer-by-layer method carried out at room temperature. For this purpose, HA (0.5 g) was suspended in an ethanolic solution of H_3btc (10 mL, 6.66 mM) and the mixture was stirred for 1 h. Afterwards, the solid was centrifuged and washed with ethanol (1 × 10 mL). The resulting material was then soaked in an ethanolic solution of $Cu(OAc)_2 \cdot H_2O$ (10 mL, 10 mM) for 1 h. Similarly, the resulting product was centrifuged and washed with ethanol (1 × 10 mL). This procedure, which represents one cycle of the layer-by-layer process, was repeated 10 times. Finally, the composite was dried at 60 ºC overnight.

Neglecting the presence of Cu_2O, the chemical composition of the composite was calculated as 0.04 mol Cu-HKUST-1@1 mol HA based on ICP-OES and TGA studies.

ICP-OES: Cu = 7.10 wt%, Ca = 17.75 wt%, P = 10.70 wt%.

TGA residue (0.04 mol 3 CuO@1 mol $Ca_{10}(PO_4)_6(OH)_2$) (calc./exp.): 98.6/99.6%.

3. X-ray absorption studies

The X-ray absorption spectra were recorded at the National Synchrotron Radiation Centre SOLARIS in Cracow, Poland, at the bending magnet PEEM/XAS beamline within 200 – 2000 eV for O (500–550 eV) K-edge as well as for Ca (300–400 eV) and Cu (910–1000 eV) L-edge. The phosphorus K (1s), L_2 and L_3-edges transitions at 2145, 136 and 135 eV, respectively, are not observed on the synchrotron at the SOLARIS centre because they are outside the experimental range. The X-ray absorption spectra were performed for Cu-HKUST-1, HA, Cu-HKUST-1@HA, and the composite incubated in DPBS solution for 8 h, 72 h, and 21 d, respectively. Samples were finely ground and attached to double-sided adhesive conductive graphite tape. The step size of 0.2 eV was used for all measurements for the edge regions and 0.4 eV for the remaining regions. Attenuation lengths for photons in the soft X-ray regime typically range between 0.1 µm to 1 µm in the 400 eV to 2000 eV energy range. Above 2000 eV, attenuation lengths can grow up to hundreds of microns, while below 400 eV they can approach 0.01 µm. The data sets were collected at room temperature under ultra-high vacuum (UHV) in the total electron yield mode (TEY). The data were processed using the PyMca 5.4.0 program package and were evaluated using the Origin Pro program as the data analysis and graphing software.

4. Nanomechanical properties

In order to determine the nanomechanical properties, the test materials were applied to a polished Ti13Zr13Nb titanium alloy surface with a roughness of 0.13 µm. To 0.1 g of each material, 1 mL of ethanol (99.8%, Sigma Aldrich) was added, then pipetted and subjected to shaking using a laboratory shaker (1500 rpm for 20 s). The colloidal suspensions thus prepared were applied to the titanium surfaces and allowed to evaporate.

To determine the nanomechanical properties, a nanoindenter (NanoTest Vantage, MicroMaterials, The United Kingdom) with a Berkovich diamond indenter was used. Ten independent measurements were performed on each sample with a maximum force of 1000 µN in loading time 40 s, hold with a maximum force of 5 s, and unloading time of 30 s. The distance between indents was 20 µm. After each indentation test, temperature drift was allowed for 10 s. The nanohardness reduced Young's modulus, plastic work, and elastic work using Oliver-Pharr methods were determined. To obtain Young`s modulus from the reduced Young's modulus values, the Poisson's ratio for the tested materials was assumed to equal 0.28.

5. Buffer stability tests

Stability test for Cu-HKUST-1@HA was determined in a physiological-like buffered (pH = 7.4) solution (DPBS). For this purpose equal amounts of composite in DPBS at a concentration of 1.5 mg/mL in clean and sterile polyvinyl chloride bottles were placed inside an incubator at a controlled temperature of 37 ºC under continuous stirring (100 rpm) for 4, 8, 24 and 72 hours, and 7, 14, 21 days. At each selected time, the powder was removed from the DPBS by filtration, and the supernatants were collected. The powder was washed with distilled water (3 × 5 mL), and dried at 50 ºC for 24 h. The stability of the composite was determined by PXRD and IR spectroscopy. Changes in the composition of the powder during 3 weeks of incubation were monitored by ICP-OES. In addition, the leaching of Ca, P and Cu in supernatants was determined using ICP-OES.

6. *In vitro* cytotoxicity assay

The *in vitro* cytotoxicity was assessed by using a human dermal fibroblast (HDF) cell line. HDF cells were purchased from Biokom (Poland). The cells were grown in DMEM-LG (Dulbecco's Modified Eagle's Medium, Low Glucose) medium containing 10% FBS (Fetal Bovine Serum) and 1% penicillin/streptomycin solution at 37 ºC in a humidified atmosphere with 5% CO_2. A volume of 5 µL containing approximately 1×10^4 cells was seeded to each well of a 96-well plate 24 h before the experiments started. For cytotoxicity evaluation, the tested materials were added to the growing cells in concentrations of 1, 10, 100, 500, 1000 and 5000 µg/mL and incubated for the next 24 h. Subsequently, an MTT (3-(4,5-dimethylthiazol-2-yl)-2,5-diphenyltetrazolium bromide) test based on the ability to reduce MTT by mitochondrial dehydrogenases was performed in triplicate to assess the cell metabolic activity (viability).

7. Hemolytic activity

The hemolytic activity of Cu-HKUST-1@HA was determined based on the procedure reported by Jaromin et al.[7] approved by the Bioethics Commission at the Lower Silesian Medical Chamber (1/PNHAB/2018). Briefly, freshly isolated human erythrocytes were transferred to the test tubes containing Cu-HKUST-1@HA (50, 100, 250, 500 and 1000 µg/mL) and PBS buffer. Subsequently, samples were mixed and incubated at 37 ºC for 30 min. Control samples were also prepared - negative (erythrocytes in PBS buffer) and positive (erythrocytes in double distilled water), respectively. Finally, after centrifugation, the absorbance of released hemoglobin was determined at 540 nm. All experiments were performed in six replicates.

8. Antimicrobial activity

Cu-based MOFs: Cu-HKUST-1, $[Cu(Hdmpzc)_2]$, $[Cu(2\text{-pymo})_2]_n$ and $NH_4[Cu_3(\mu_3\text{-OH})(\mu_3\text{-4-carboxypyrazolato})_3]$), hydroxyapatite (HA) and Cu-HKUST-1@HA composite were evaluated for antimicrobial activities against Gram-positive (*Staphylococcus aureus* ATCC 25923) and Gram-negative (*E. coli* ATCC 25922, *Klebsiella pneumoniae* ATCC 700603, *Pseudomonas aeruginosa* ATCC 10145) bacteria and against yeast of *Candida albicans* ATCC 10231. Microbial strains were purchased from the Leibniz Institute DSMZ-German Collection of Microorganisms and Cell Cultures GmbH in Braunschweig, Germany. The antimicrobial efficacy was examined using the standard broth dilution method according to the Clinical and Laboratory Standards Institute (CLSI). The assay was performed in triplicate using 96-well plates. The final concentration of microorganisms in each well of the plate was 5×10^5 c.f.u. mL^{-1}. The Tripticase Soy Broth (TSB, Becton Dickinson) and Sabouraud Dextrose Broth (SDB, Becton Dickinson) were used for bacterial and yeast growth, respectively. The final volume of the sample (medium with MOFs, HA or Cu-HKUST-1@HA and microorganisms) in each well was 150 μL. Compounds were tested in a concentration range from 1 to 10 mg/mL (at intervals of 1 mg). The positive controls contained corresponding broth medium inoculated with microorganisms, while the negative control contained non-inoculated broth. Inoculated plates were incubated for 24 h at 37 ºC in shaking conditions (100 rpm). The antimicrobial efficacy was expressed as the minimum inhibitory concentration (MIC) that inhibited the growth of microorganisms (estimated visually). Minimum biocidal concentrations (MBCs) were also determined after spreading of 100 μL of each test sample on the surface of TSA (for bacteria) or SDA (for *C. albicans*) in Petri plates and incubation for 24 h at 37 ºC. The MBCs of tested compounds were defined as the lowest concentration of antimicrobial agent that prevented the growth of $> 99.9\%$ microbial cells.

9. Additional figures

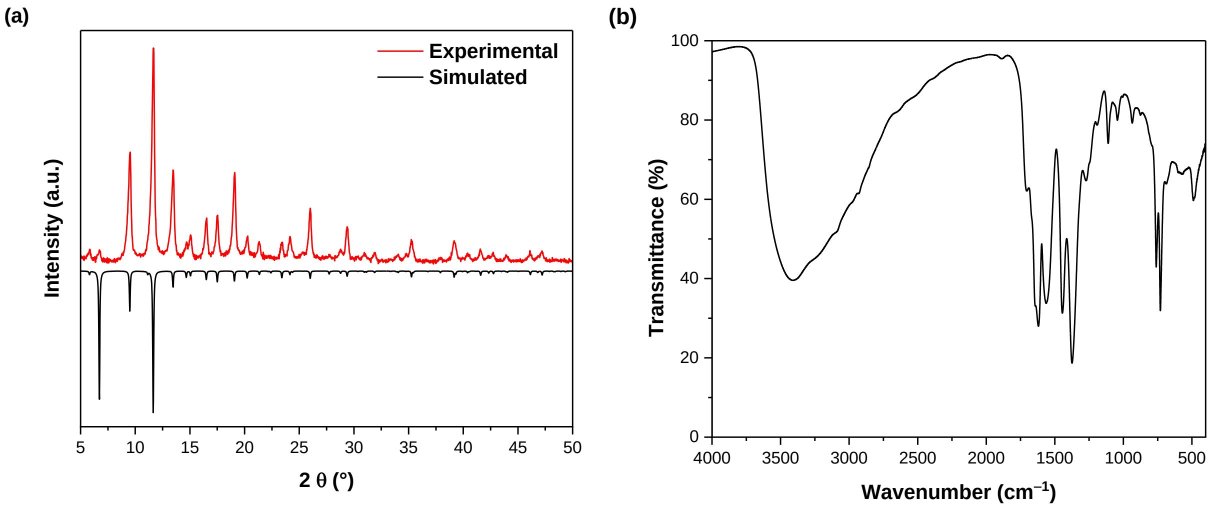

Figure S1. Powder X-ray diffraction patterns (a) and IR spectrum (b) of Cu-HKUST-1.

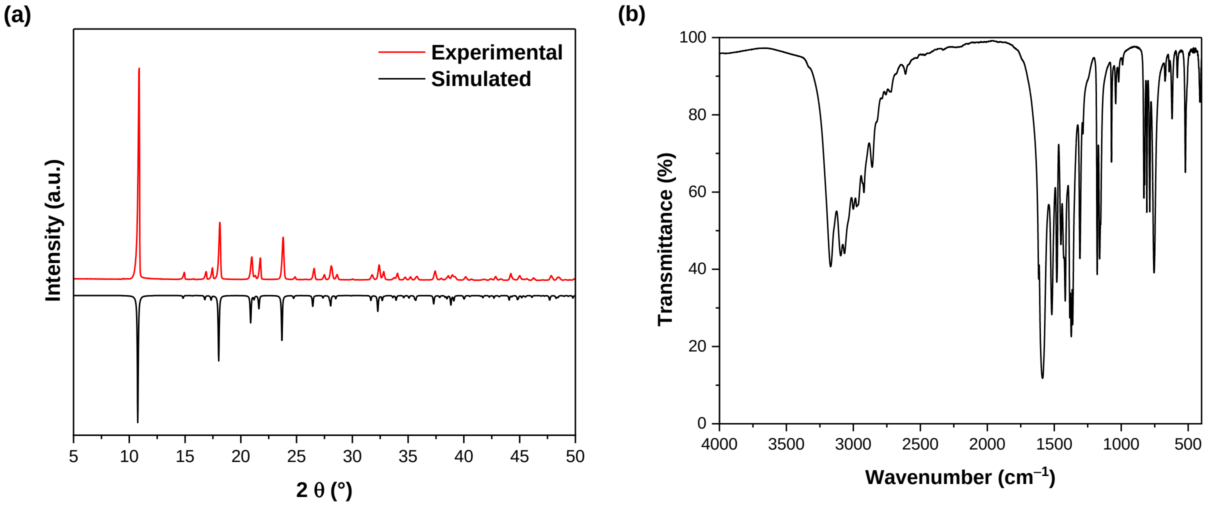

Figure S2. Powder X-ray diffraction patterns (a) and IR spectrum (b) of $[Cu(Hdmpzc)_2]$.

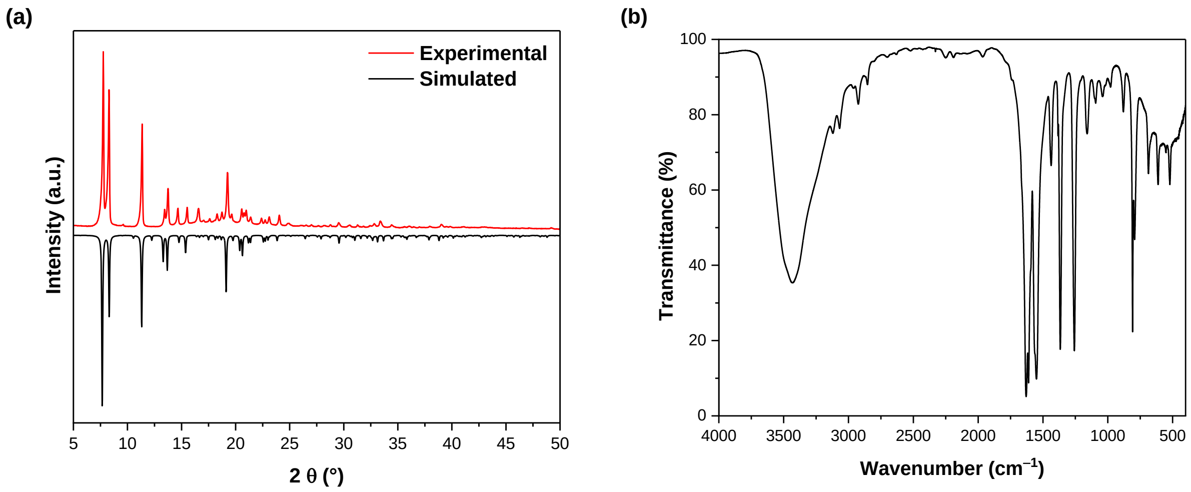

Figure S3. Powder X-ray diffraction patterns (a) and IR spectrum (b) of $[Cu(2\text{-pymo})_2]_n$.

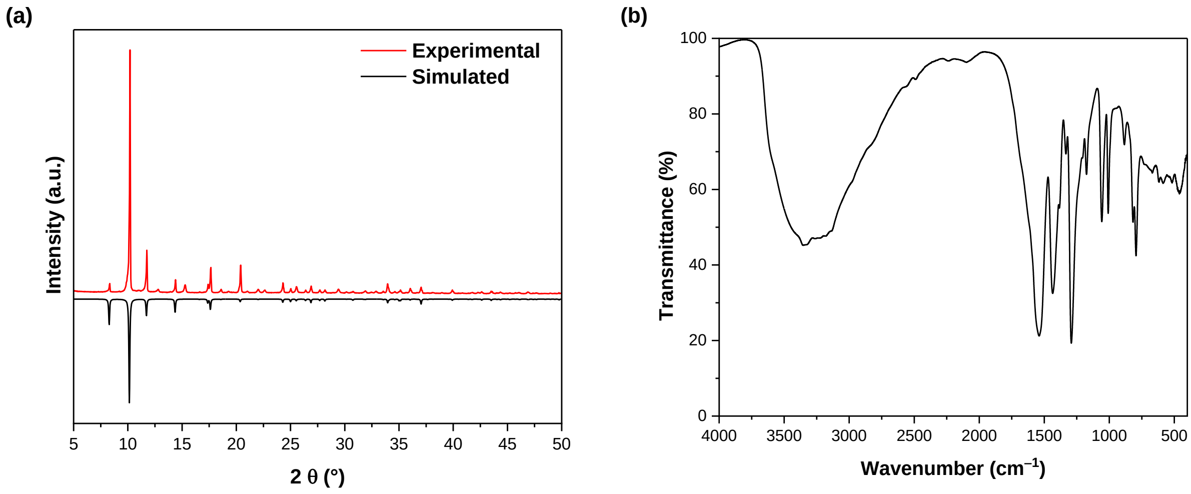

Figure S4. Powder X-ray diffraction patterns (a) and IR spectrum (b) of $NH_4[Cu_3(\mu_3\text{-}OH)(\mu_3\text{-}4\text{-}carboxypyrazolato)_3$.

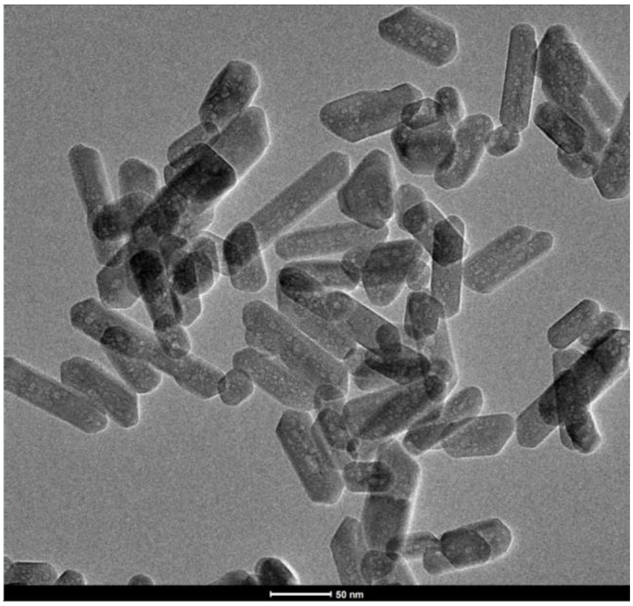

Figure S5. TEM image of HA nanorods.

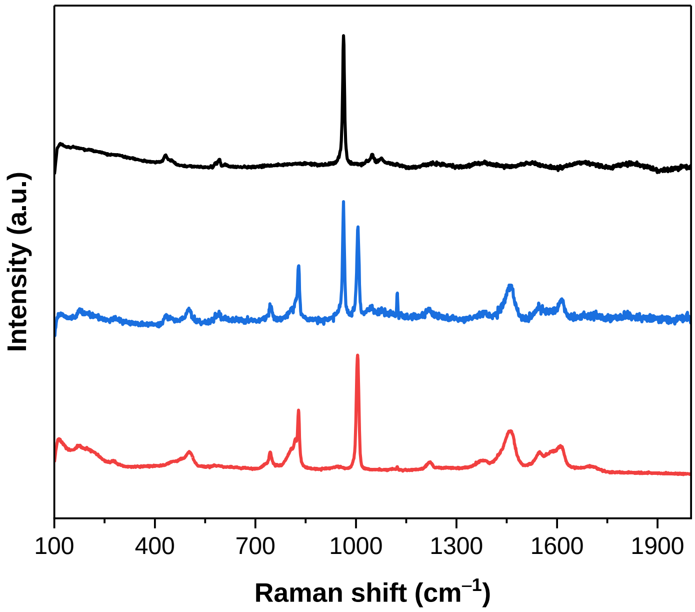

Figure S6. Raman spectra of Cu-HKUST-1 (red), Cu-HKUST-1@HA (blue) and HA (black).

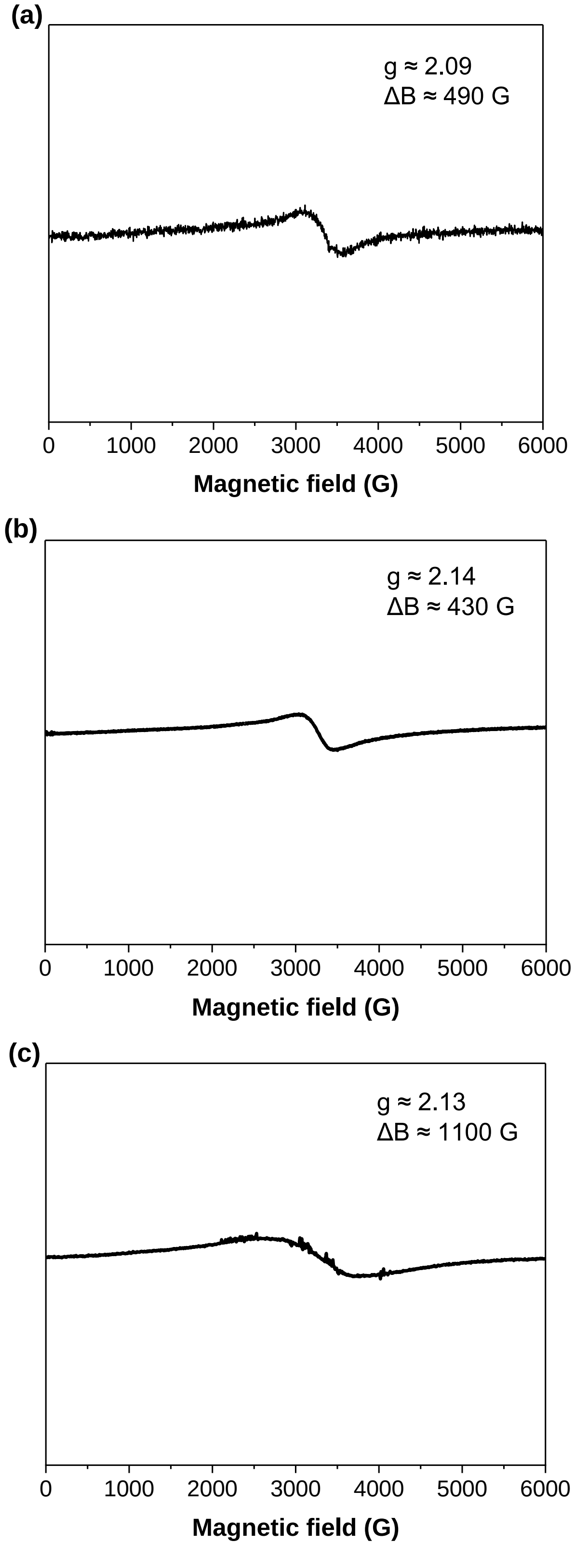

Figure S7. EPR spectra of Cu-HKUST-1@HA (a), Cu-HKUST-1_mech (b) and Cu-HKUST-1_solv at RT with EPR parameters inserted.

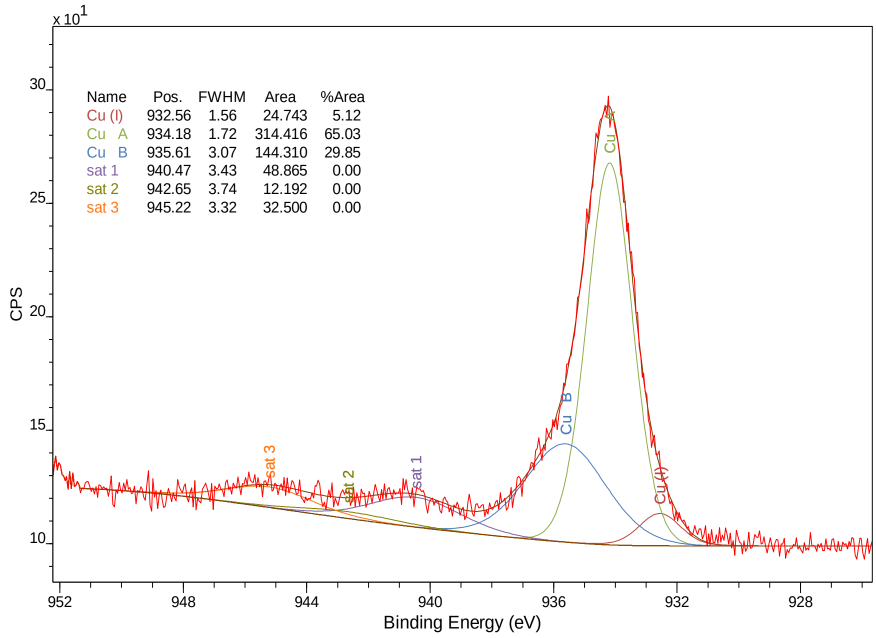

Figure S8. The high-resolution Cu 2p XPS spectra of Cu-HKUST-1@HA.

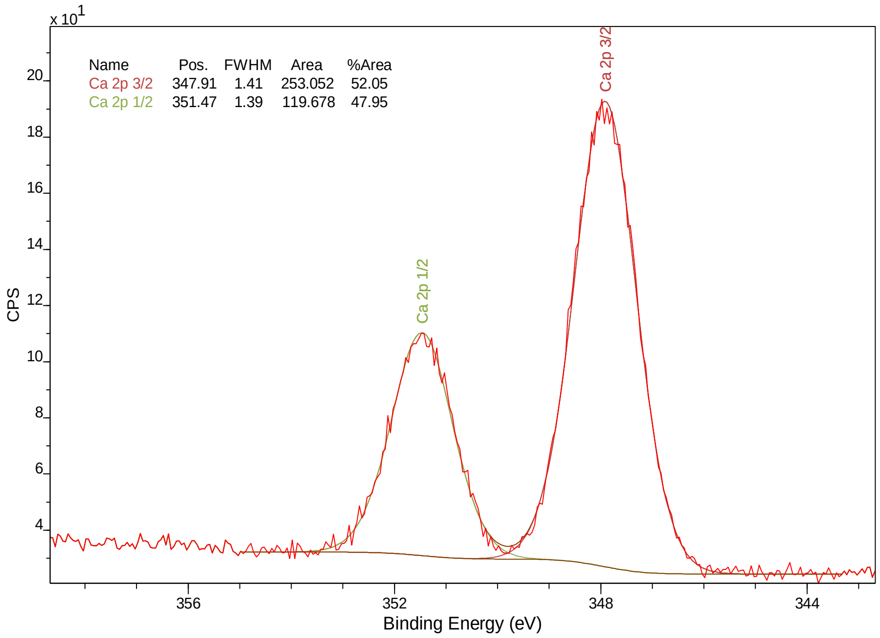

Figure S9. The high-resolution Ca 2p XPS spectra of Cu-HKUST-1@HA.

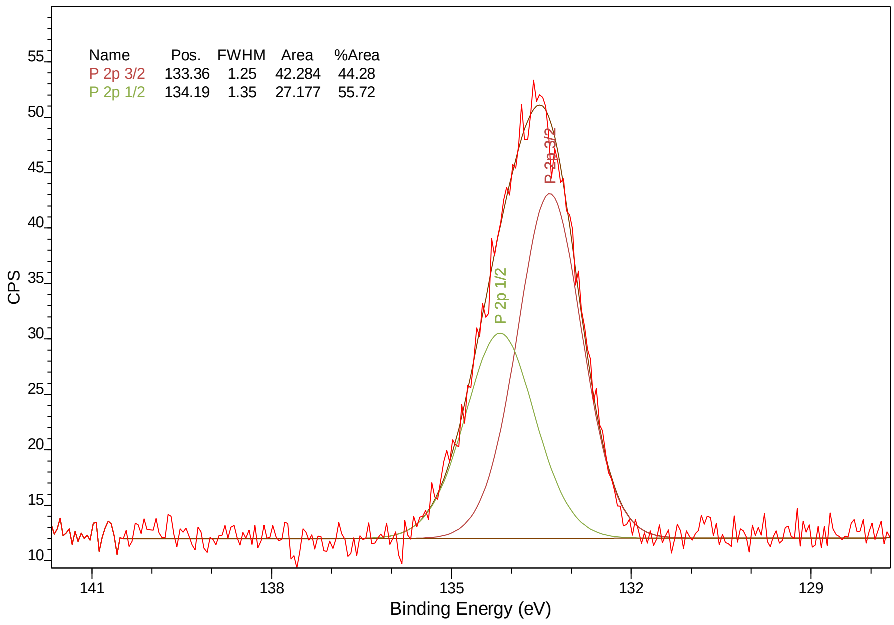

Figure S10. The high-resolution P 2p XPS spectra of Cu-HKUST-1@HA.

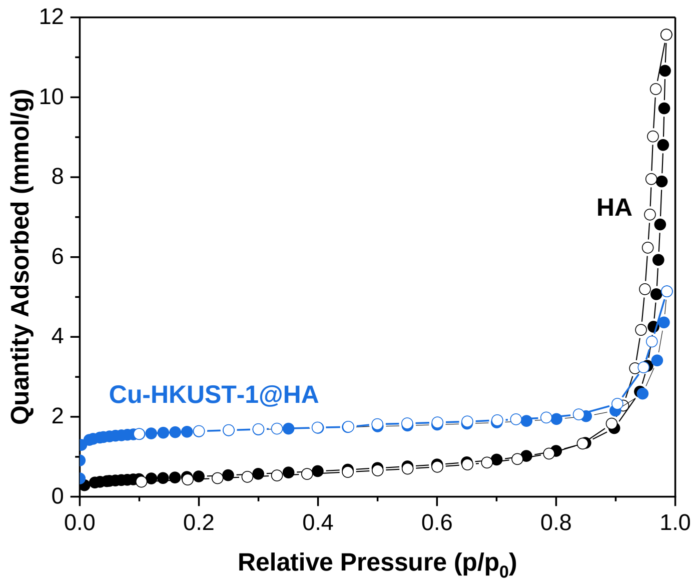

Figure S11. N_2 adsorption (filled symbols)-desorption (empty symbols) isotherms measured at 77 K for HA and Cu-HKUST-1@HA.

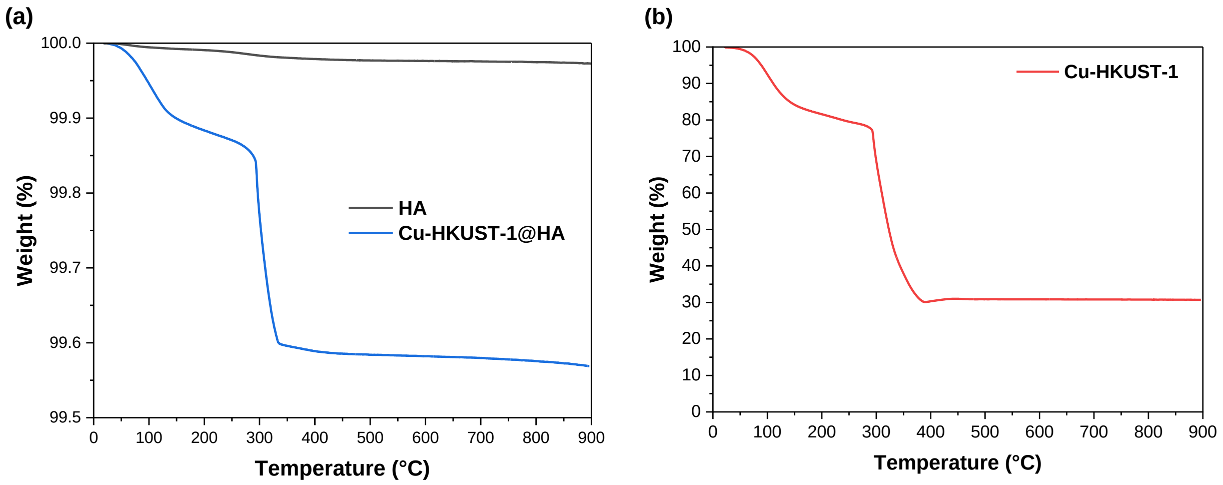

Figure S12. TGA curves of HA and Cu-HKUST-1@HA (a), Cu-HKUST-1 (b).

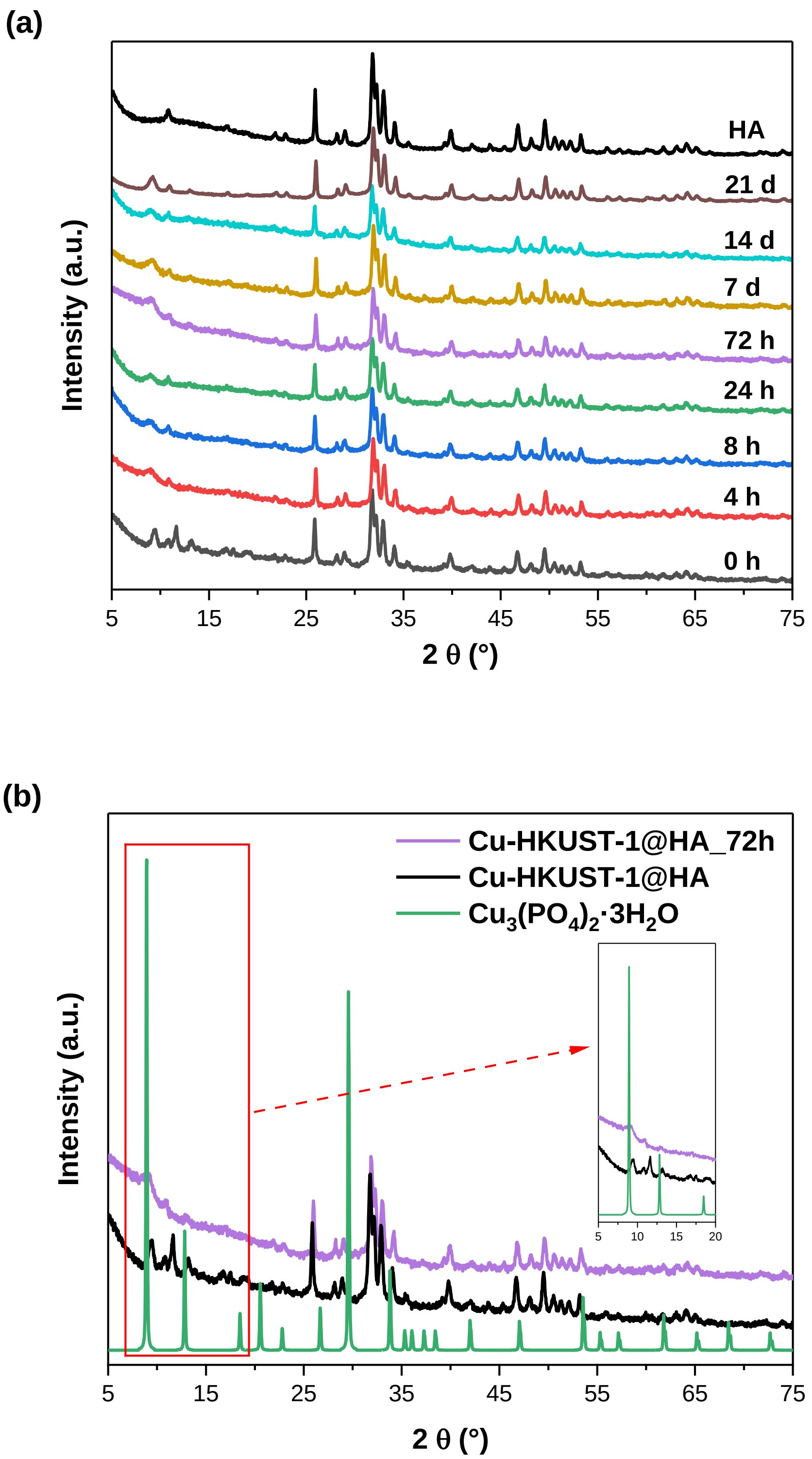

Figure S13. Powder X-ray diffraction patterns of (a) Cu-HKUST-1@HA before and after incubation in DPBS buffer (37 ºC, 4 hours – 21 days); (b) standard $Cu_3(PO_4)_2{\cdot}3H_2O$ (PDF-2 00-001-0054) along with Cu-HKUST-1@HA before and after selected incubation time in DPBS buffer (37 ºC, 72 h).

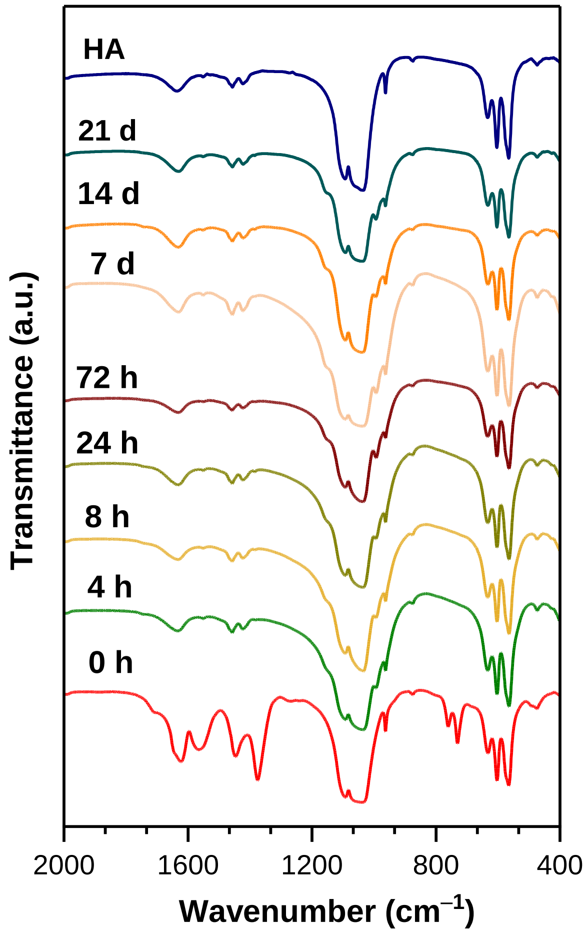

Figure S14. IR spectra of Cu-HKUST-1@HA before (0 h) and after incubation in DPBS buffer (37 ºC, 4 hours – 21 days).

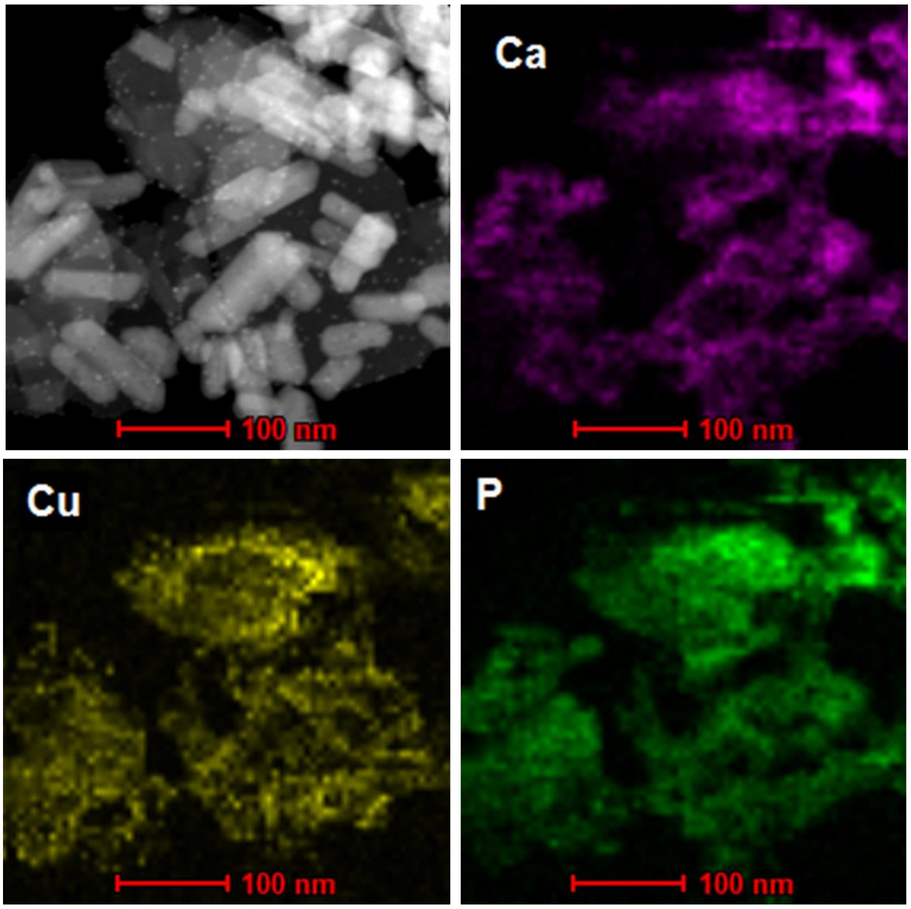

Figure S15. STEM-EDS analysis of Cu-HKUST-1@HA after 21 days of incubation in DPBS (37 ºC).

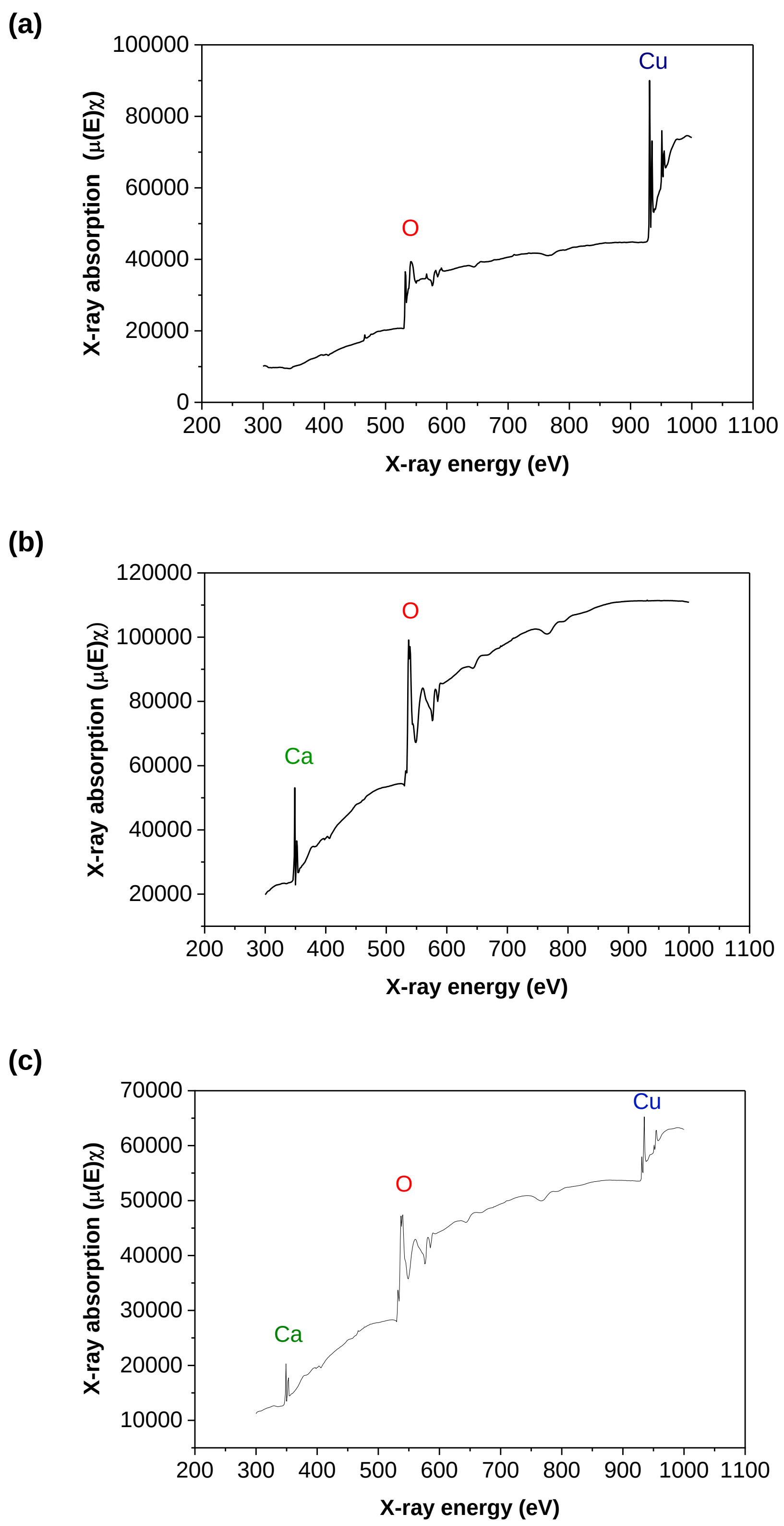

Figure S16. The XAS spectra of Cu-HKUST-1 (a), HA (b), and Cu-HKUST-1@HA (c).

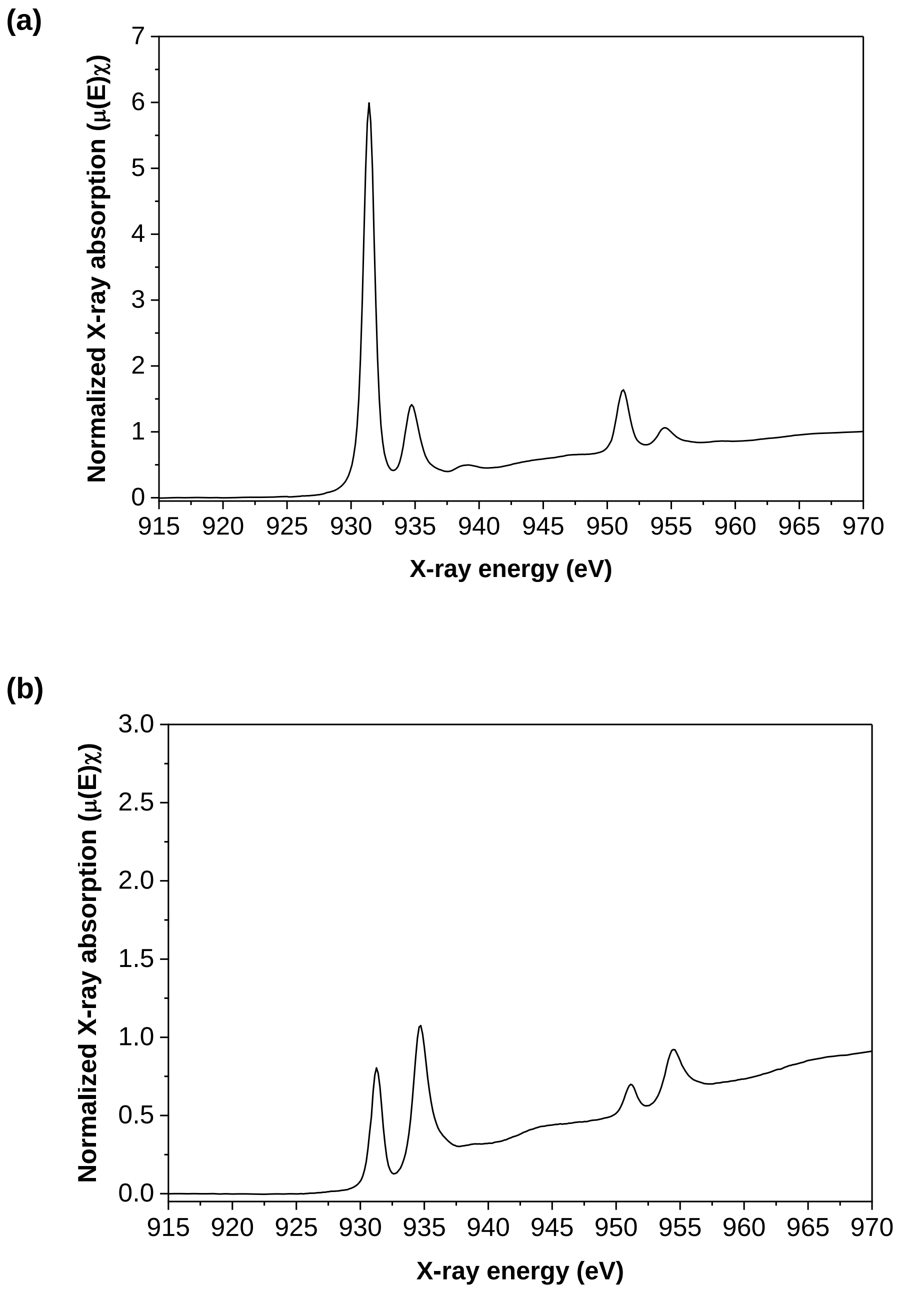

Figure S17. Normalized Cu L-edge absorption spectra of Cu-HKUST-1 (a), Cu-HKUST-1@HA (b).

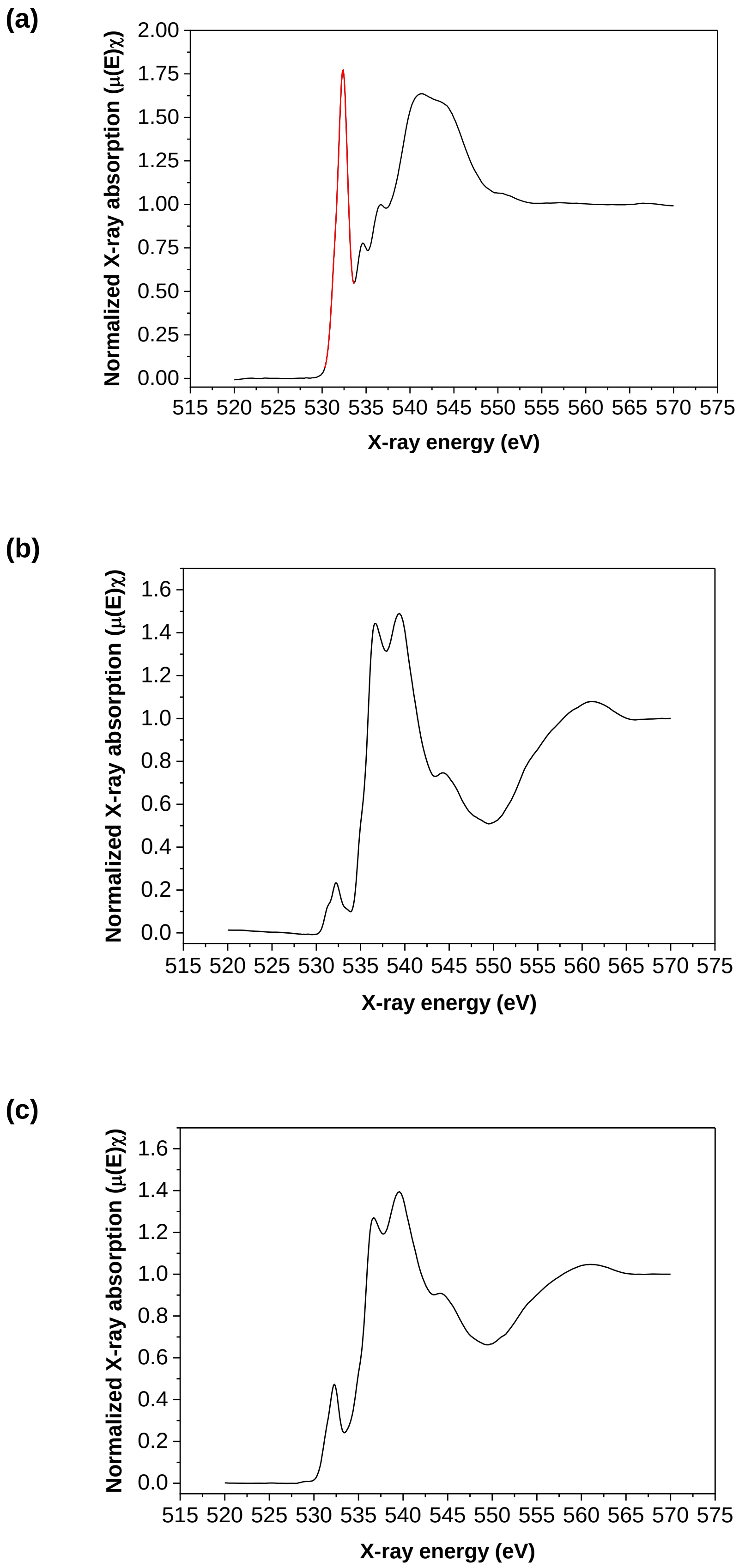

Figure S18. Normalized O K-edge absorption spectra of the Cu-HKUST-1 (a), HA (b), and Cu-HKUST-1@HA (c).

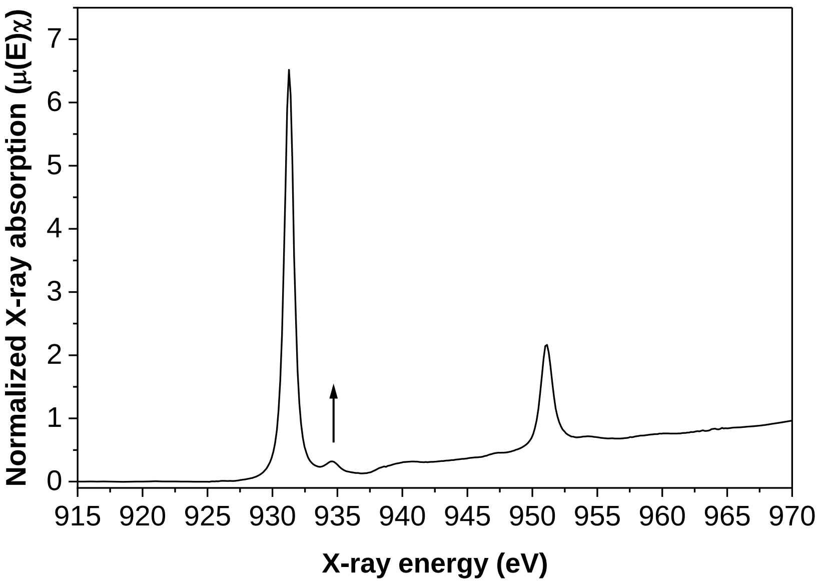

Figure S19. Normalized Cu L-edge absorption spectra of Cu-HKUST-1@HA after incubation in DPBS for 21 days.

(a)

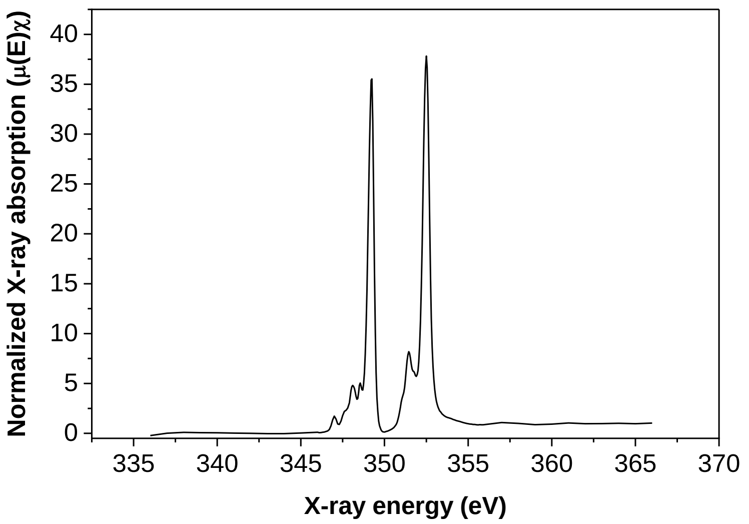

Normalized X-ray absorption (μ(E)χ)
0
5
10
15
20
25
30
35
40
335
340
345
350
355
360
365
370
X-ray energy (eV)

(b)

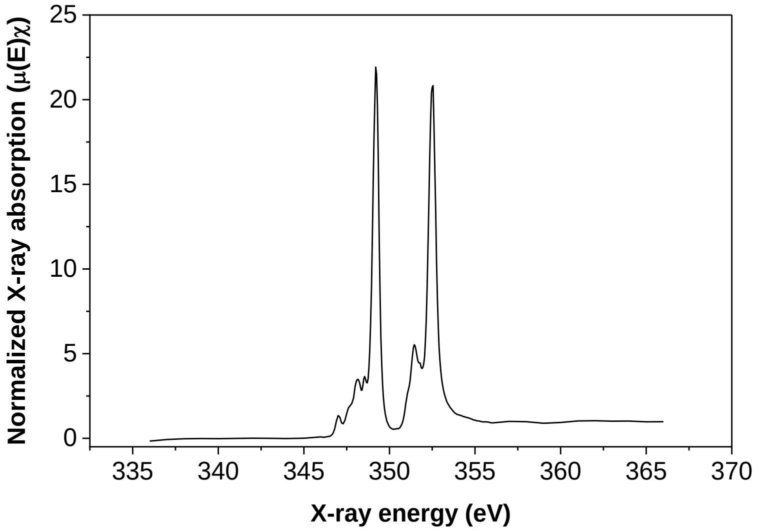

Normalized X-ray absorption (μ(E)χ)
0
5
10
15
20
25
335
340
345
350
355
360
365
370
X-ray energy (eV)

(c)

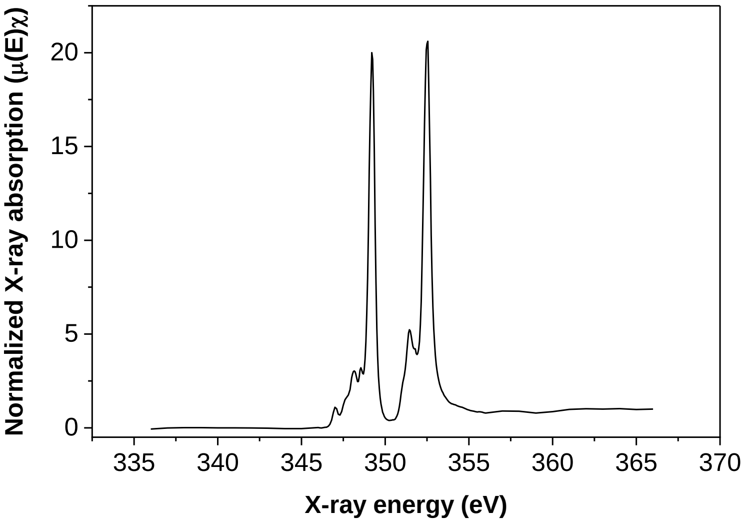

Normalized X-ray absorption (μ(E)χ)
0
5
10
15
20
335
340
345
350
355
360
365
370
X-ray energy (eV)

(d)

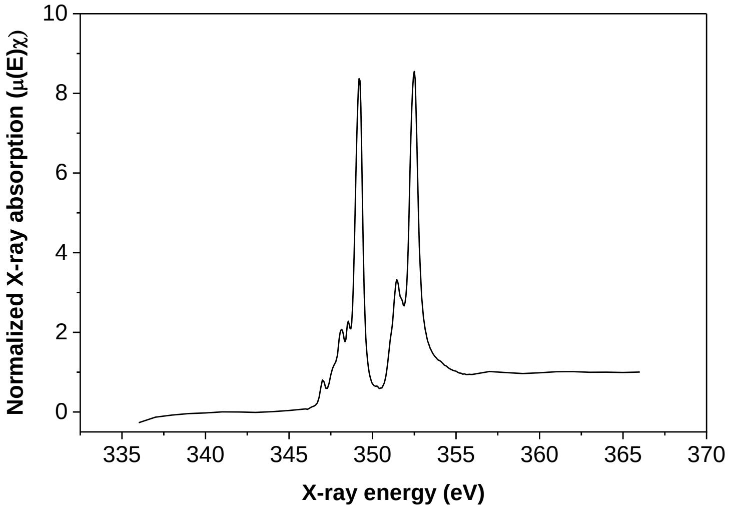

(e)

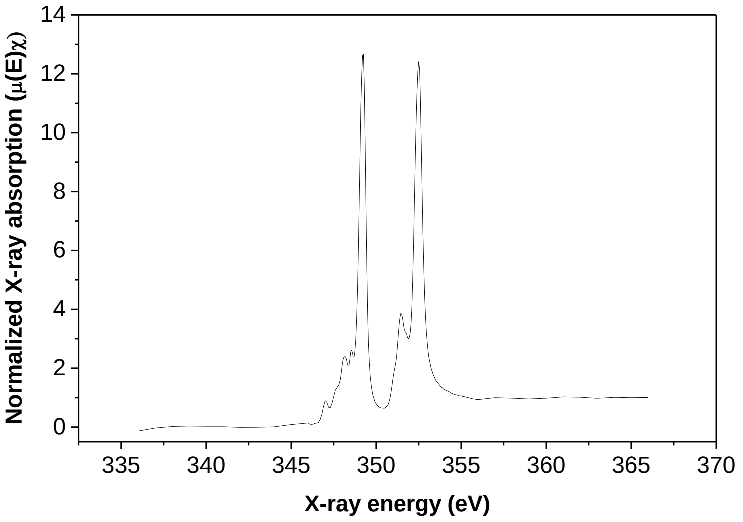

Figure S20. Normalized Ca L-edge absorption spectra of HA (a), Cu-HKUST-1@HA (b), Cu-HKUST-1@HA incubated in DPBS for 8 h (c), 72 h (d), and 21d (e), respectively.

10. Bibliography

(1) Silvia Bordiga, Laura Regli, Francesca Bonino, Elena Groppo, Carlo Lamberti, Bo Xiao, Paul S. Wheatley, Russell E. Morris and Adriano Zecchina, Adsorption properties of HKUST-1 toward hydrogen and other small molecules monitored by IR. *Phys. Chem. Chem. Phys.,* **2007**, 9, 2676–2685.

(2) Timothy Steenhaut, Nicolas Grégoire, Gabriella Barozzino-Consiglio, Yaroslav Filinchuk, and Sophie Hermans. Mechanochemical defect engineering of HKUST-1 and impact of the resulting defects on carbon dioxide sorption and catalytic cyclopropanation. *RSC Adv.* **2020**, 10, 19822–19831.

(3) Aurel Tăbăcaru, Nertil Xhaferaj, Luísa M. D. R. S. Martins, Elisabete C. B. A. Alegria, Rogério S. Chay, Carlotta Giacobbe, Konstantin V. Domasevitch, Armando J. L. Pombeiro, Simona Galli, and Claudio Pettinari. Metal Azolate/Carboxylate Frameworks as Catalysts in Oxidative and C-C Coupling Reactions. *Inorg. Chem.* **2016**, 55, 12, 5804–5817.

(4) Leandro C. Tabares, Jorge A. R. Navarro, and Juan M. Salas. Cooperative Guest Inclusion by a Zeolite Analogue Coordination Polymer. Sorption Behavior with Gases and Amine and Group 1 Metal Salts. *J. Am. Chem. Soc.* **2001**, 123, 3, 383–387.

(5) Elsa Quartapelle Procopio, Fatima Linares, Carmen Montoro, Valentina Colombo, Angelo Maspero, Elisa Barea, and Jorge R. Navarro. Cation-Exchange Porosity Tuning in Anionic Metal–Organic Frameworks for the Selective Separation of Gases and Vapors and for Catalysis. *Angew. Chemie Int. Ed.* **2010**, 49, 7308–7311.

(6) Marzena Fandzloch, Carmen R. Maldonado, Jorge A. R. Navarro, and Elisa Barea, Biomimetic 1-Aminocyclopropane-1-Carboxylic Acid Oxidase Ethylene Production by MIL-100(Fe)-Based Materials. *ACS Appl. Mater. Interfaces* **2019**, 11, 37, 34053–34058.

(7) Anna Jaromin, Mariola Korycińska, Magdalena Piętka-Ottlik, Witold Musiał, Wanda Peczyńska-Czoch, Łukasz Kaczmarek, and Arkadiusz Kozubek. Membrane perturbations induced by new analogs of neocryptolepine. *Biol Pharm Bull.* **2012**, 35, 9, 1432–1439.
